# Endometriosis MR mimickers: T1-hyperintense lesions

**DOI:** 10.1186/s13244-023-01587-3

**Published:** 2024-01-24

**Authors:** Edouard Ruaux, Stéphanie Nougaret, Marie Gavrel, Mathilde Charlot, Mojgan Devouassoux-Shisheboran, François Golfier, Isabelle Thomassin-Naggara, Pascal Rousset

**Affiliations:** 1 Department of Radiology, Hospices Civils de Lyon, Lyon Sud University Hospital, Lyon 1 Claude Bernard University, 165 Chemin du Grand Revoyet, EMR 3738, 69495 Pierre Bénite, France; 2 Department of Radiology, Montpellier Cancer Institute, U1194, Montpellier University, 34295 Montpellier, France; 3 Department of Radiology, Hospices Civils de Lyon, Lyon Sud University Hospital, Lyon 1 Claude Bernard University, EMR 3738, Pierre Bénite, France; 4 Department of Pathology, Hospices Civils de Lyon, Lyon Sud University Hospital, Lyon 1 Claude Bernard University, 69495 Pierre Bénite, France; 5 Department of Gynecology and Obstetrics, Hospices Civils de Lyon, Lyon Sud University Hospital, Lyon 1 Claude Bernard University, EMR 3738, 69495 Pierre Bénite, France; 6 Department of Radiology, Service Imageries Radiologiques et Interventionnelles Spécialisées, Hôpital Tenon, Assistance Publique Hôpitaux de Paris, Sorbonne Université, 75020 Paris, France

**Keywords:** Endometriosis, Deep infiltrative endometriosis, Ovarian cysts, Functional hemorrhagic cysts, Magnetic resonance imaging

## Abstract

Endometriosis is a chronic and disabling gynecological disease that affects women of reproductive age. Magnetic resonance imaging (MRI) is considered the cornerstone radiological technique for both the diagnosis and management of endometriosis. While MRI offers higher sensitivity compared to ultrasonography, it is prone to false-positive results, leading to decreased specificity. False-positive findings can arise from various T1-hyperintense conditions on fat-suppressed T1-weighted images, resembling endometriotic cystic lesions in different anatomical compartments. These conditions include hemorrhage, hyperproteic content, MRI artifacts, feces, or melanin. Such false positives can have significant implications for patient care, ranging from incorrect diagnoses to unnecessary medical or surgical interventions and subsequent follow-up. To address these challenges, this educational review aims to provide radiologists with comprehensive knowledge about MRI criteria, potential pitfalls, and differential diagnoses, ultimately reducing false-positive results related to T1-hyperintense abnormalities.

**Critical relevance statement**

MRI has a 10% false-positive rate, leading to misdiagnosis. T1-hyperintense lesions, observed in the three phenotypes of pelvic endometriosis, can also be seen in various other causes, mainly caused by hemorrhages, high protein concentrations, and artifacts.

**Key points**

• MRI in endometriosis has a 10% false-positive rate, leading to potential misdiagnosis.

• Pelvic endometriosis lesions can exhibit T1-hyperintensity across their three phenotypes.

• A definitive diagnosis of a T1-hyperintense endometriotic lesion is crucial for patient management.

• Hemorrhages, high protein concentrations, lipids, and artifacts are the main sources of T1-hyperintense mimickers.

**Graphical Abstract:**

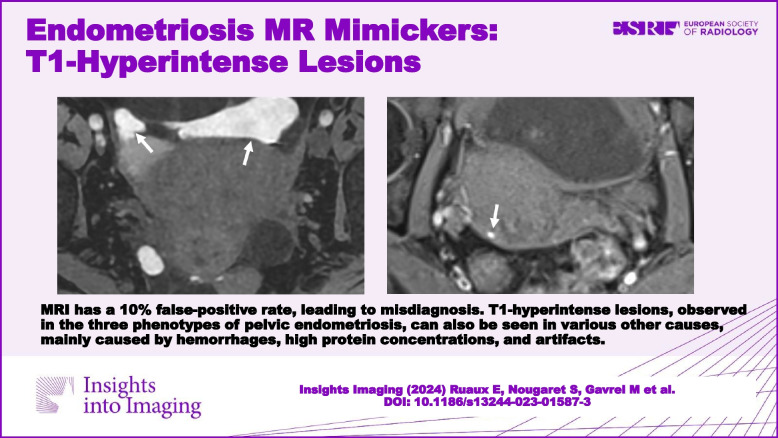

**Supplementary Information:**

The online version contains supplementary material available at 10.1186/s13244-023-01587-3.

## Background

Pelvic endometriosis is a chronic inflammatory disease defined by the presence of endometrial-like tissue outside the uterine cavity. It is estimated to affect 5 to 10% of women of reproductive age [1]. Common symptoms experienced by patients with endometriosis include chronic pelvic pain, dysmenorrhea, dyspareunia, dyschezia, dysuria, and infertility, depending on the anatomic location and degree of infiltration, which can greatly affect quality of life [2]. Endometriosis is divided into three clinical phenotypes that can coexist: ovarian cysts that known as endometriomas, superficial peritoneal endometriotic implants, and deep infiltrative endometriosis (DIE) [3]. Hemorrhagic changes are commonly observed in all these phenotypes. Endometriomas occur when ectopic endometrial-like tissue implants invaginate into the ovarian parenchyma, leading to cyclical bleeding in response to hormonal stimulation and resulting in hemorrhagic ovarian cysts. This is the most prevalent form of endometriosis observed in magnetic resonance imaging (MRI) [4]. Superficial endometriotic implants are rarely visible on imaging. Therefore, they can be identified based on their microcystic and/or hemorrhagic nature. DIE is defined as the extension of endometrial-like tissue beneath the peritoneal surface, with the ability to invade surrounding structures. It is associated with fibrosis, hemorrhagic changes, and disruption of normal anatomical structures.

This disease represents a public health issue [5], impacting patients’ quality of life. Thus, the importance of accurate and early diagnosis must be emphasized. Transvaginal ultrasound is the first-line imaging modality for initial evaluation and management [6]. However, MRI has the highest accuracy, which aids in accurate diagnosis, and provides a detailed mapping of the extent and severity of the disease. The more detailed information obtained from MRI results improves the quality of care gynecologists can provide when considering medical and surgical management with patients [7]. The overall sensitivity of MRI in diagnosing endometriosis is estimated at 94% [8]; however, the specificity is slightly lower, leading to a 10% false-positive rate [9]. This limitation underscores the importance of educating radiologists about potential causes of inaccurate MRI results. In the dedicated recommended MRI protocol [10], fat-suppressed T1-weighted images (WI) is a pivotal sequence that allows for the detection of cystic endometriotic hemorrhagic component, whatever the phenotype of lesion [10]. However, various degrees of T1-hyperintense cystic lesions may be observed within the pelvis due to other physical entities, such as hemorrhage from other causes, high concentrations of proteins, lipids, melanin, feces, or artifacts. These sources of false positives can significantly impact patient management. Misdiagnosing endometriosis during the initial diagnosis, or even in the event of incidental discovery on pelvic MRI can lead to inappropriate medical treatment up to unnecessary surgery and severe psychological consequences for the patient. In cases of confirmed pelvic endometriosis, mistaking other T1-hyperintense findings for endometriosis may overestimate the extent and the severity of the disease leading to inappropriate decision-making choice between medical and surgical treatments.

Thus, the aim of this review is to provide guidance to radiologists so that they may better differentiate T1-hyperintense endometriotic lesions from other pelvic lesions and imaging artifacts. We will highlight the importance of multi-sequence reading, morphological analysis, and accurate anatomical localization, as these factors can enhance diagnostic accuracy and improve patient care.

## T1-hyperintense typical features of pelvic endometriosis

**Fig. 1:**
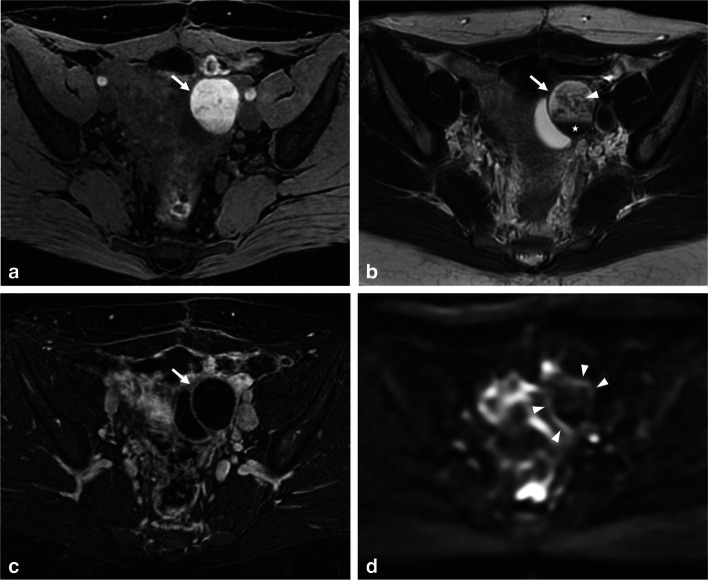
Functional hemorrhagic ovarian cyst in a 28-year-old woman with dysmenorrhea and acute pelvic pain at the time of MRI, and no medical history. **a** Axial T1-W fat-suppressed MR image shows a T1-hyperintense left hemorrhagic ovarian cyst (arrow). **b** Axial T2-W MR image shows a heterogeneous cyst (arrow) with upper hypointense lace-like reticular areas (arrowhead) and lower fluid-fluid level (star) with T2 shading. **c** Axial T1-W contrast-enhanced subtracted MR image shows homogeneous hyperintense rim wall enhancement (arrow). **d** Axial diffusion-weighted MR image shows an equivalent hyperintense “DWI-ring sign” (arrowheads) compared to **c**

Endometriomas appear as cystic ovarian structures (> 10 mm) containing degenerated blood from repeated cyclic hemorrhage, resulting in T1-hyperintensity due to methemoglobin, that markedly shorten the T1 of fluids [11]. Further, low T2 signal intensity, also known as shading, is observed due to iron overload, high protein concentrations, and high viscosity due repeated bleeding and degradation of old blood products [12]. Both a hypointense peripheral ring from hemosiderin staining and a clot-induced T2-dark spot adjacent to the cyst can be seen [13]. In rare cases (< 5%), endometriomas may appear T2-hyperintense, depending on protein and hemosiderin concentrations as well as the patient’s age [14]. Endometriomas typically have thin walls with inconsistent, absent, or low wall enhancement [15]. The ovarian-adnexal reporting and data system on magnetic resonance imaging (O-RADS MRI) score has entered clinical practice in terms of reporting ovarian lesions and indicating the possibility of malignancy [16]. Endometriomas are rated O-RADS MRI 2 (almost certainly benign) [17]. Adhesions to the surrounding anatomical structures, additional ovarian endometriotic implants (≤ 5 mm) or micro-ovarian endometriomas (< 10 mm), and bilateral endometriomas with multiplicity are also strongly suggestive of endometriosis, sometimes leading to a “kissing-ovaries” sign (Supplemental - Figure 1) [18]. DIE can affect almost any organ or structure, although most lesions are found in the pelvic cavity, particularly in the posterior compartment. DIE consists of lesions comprised of glandular, stromal, and fibrotic tissue [19], readily identifiable on MRI as T2-hypointense fibrotic lesions. Such lesions often contain microcystic changes, with variations in the number of T2-hyperintense and T1-hyperintense hemorrhagic foci dependent on ectopic glandular tissue activity. Superficial endometriotic implants are histologically defined as peritoneal implants and commonly described at surgery as bluish or clear red lesions during surgery due to hemosiderin deposition or acute hemorrhage [20]. However, due to their small size and/or low thickness, they may not be identifiable on MRI, resulting in false negatives. Despite the above, when large enough, they are well depicted as T1-hyperintense foci along the peritoneal layer or pelvic organ surface, and they may sometimes be the sole finding for the radiologist to suspect endometriosis [21].

## T1-hyperintense differential diagnosis and pitfalls for pelvic endometriosis

Various physical entities can lead to different degrees of T1-hyperintense cystic lesions in the pelvis (Table 1). Hemorrhages, high protein concentrations, lipids, and artifacts are the four primary causes of high T1-weighted (T1-W) signal intensity in various disease conditions. Of note, the systematic use of a fat-suppressed T1-weighted imaging (T1-WI) in pelvic MRI protocols is crucial to rule out differential diagnosis of fat-containing lesions [10].

**Table 1:** T1-hyperintense mimickers: MRI key features

Nature of the T1-hyperintensity	Structure involved *Type of condition*	MRI key features
**Hemorrhagic**	**Adnexa**	
	*Functional hemorrhagic cyst*	T1-hyperintense rim, « ring sign » enhancement, variable signal intensity on T2-WI, resolution on 8–12 weeks imaging follow-up on ultrasonography
	*Ovarian ischemic necrosis*	Enlarged medially displaced ovary in case of adnexal torsion, T1-hyperintense rim, T2 heterogeneous signal intensity, no enhancement of the ovary +/− fallopian tube
	*Ectopic pregnancy*	Unilateral hematosalpinx, T2-hypointense tubal debris or fetal pole , hemoperitoneum, contrast enhancement of the adnexa
	**ACUM**	Extra-ovarian topography within the uterus or the broad ligament, thick peripheral ring of muscular tissue in low signal intensity on T2-WI and with low enhancement, central round cavity with hematometra (+/− T2- shading)
**Hyperproteic**	**Adnexa**	
	*Epithelial cystic tumors*	Unilateral and unilocular thin-walled fluid-filled cyst, no shading T2-WI, papillary projections
	*Paratubal serous cyst*	Extra-ovarian with negative beak sign, no shading T2-WI, papillary projections
	*Hydrosalpinx (chronic)*	Serpentine structure, low to mild wall enhancement
	*Tubo-ovarian abscess (chronic)*	Thickened wall, hyperintense rim on T1 FS -WI, heterogeneous signal intensity on T2-WI, moderate to sustained wall enhancement
	**Vulva and vagina**	
	*Epithelial inclusion cyst*	Location within the wall or vaginal cuff depending on prior surgery or vaginal procedure, single cystic lesion, hypointense perilesional scar tissue on T2-WI in case of episiotomy
	*Gartner’s duct cyst*	Preferential location within the anterolateral wall, single thin-walled and well-defined cystic lesion, possible association with renal abnormalities
	*Bartholin’s gland cyst*	Posterolateral surface of the vestibule, can be bilateral with symmetric location
	*Skene’s gland cyst*	Along the posterior course of distal urethra, small unilocular cyst
	**Urachus**	
	*Urachal insertion cyst*	Small to middle-sized single cyst at the exact insertion of urachus, thin wall, mostly hyperintense on T2-WI, no fibromuscular component
	**Peritoneum**	
	*Multicystic peritoneal mesothelioma*	Multicystic grape-like lesions with some loculi in high signal intensity on T1 FS -WI, with no fibrous tissue, often with multi-focal peritoneal involvement
	**Retroperitoneal**	
	*Tailgut cyst*	Uni- or multilocular retrorectal cyst, variable size (mostly small) along anococcygeal raphe
**Artifacts**	**Vessels**	
	*Vascular Flow-Related Enhancement*	Any highcirculating vessels, linear or serpiginous structures on other sequences or MPR 3D T2-WI, flow-voids on T2-WI, disappearance using spatial saturation bands
	*Vascular ghosts*	Illiac vessels with ghosting in the direction of phase-encoding, not seen on other sequences
	**Calcification (phlebolith)**	Endoveinous location (mostly parametrium and paravagina), tiny round low signal intensity on T2-WI, calcium hyperdensity on CT
**Feces**	**Appendix / Sigmoid diverticula**	Endoluminal digestive connection or location on other sequences or MPR 3D T2-WI
**Melanin**	**Vulva and vagina**	Variable signal intensity on T2-WI, solid component enhancement on contrast-enhanced subtracted MR images

### Hemorrhagic T1-hyperintense cyst

The MRI signal characteristics of a hemorrhagic lesion largely depend on the age of the hemorrhage. The presence of intracellular and extracellular methemoglobin within the lesion creates a paramagnetic effect that affects T1 relaxation times. This effect leads to a shortening of T1 relaxation times, resulting in high signal intensity on MRI. The degree of T1-shortening is influenced by the duration of the hemorrhage, ranging from 3 days to several months.

#### Adnexa

##### Functional hemorrhagic cysts

Differentiating endometriomas from hemorrhagic cysts (O-RADS MRI 1) can be challenging. Both can appear as solitary, unilocular T1-hyperintense cysts with smooth linings. However, hemorrhagic cysts often show a T1-hyperintense rim due to peripheral oxidation of an acute hematoma (deoxyhemoglobin) [22]. Hemorrhagic cysts also exhibit heterogeneous and mild signal loss on T2-WI, with possible shading but lacking a T2-dark spot [13]. Contrast-enhanced imaging reveals enhancement of the cyst wall, forming a “ring sign” (better seen on post-contrast-enhanced subtracted T1-W images) [22]. Additionally, analyzing internal enhancement after contrast administration can help differentiate a clot (non-enhanced solid component) from a mural nodule (enhanced solid tissue) (Fig. 1). While diffusion-WI (DWI) is not recommended but optional in ESUR guidelines, some studies suggest that endometriomas have lower apparent diffusion coefficient values on DWI (around 1 ± 0.1 × 10^−3^ mm^2^) compared to other cystic ovarian lesions, including functional hemorrhagic cysts [23, 24]. In our experience, DWI can show a hyperintense rim, known as the “DWI-ring sign” favoring a hemorrhagic cyst. Lastly, ultrasound follow-up after a few menstrual cycles allows for definitive diagnosis, as hemorrhagic cysts tend to completely or partially resolve, while endometriomas persist [25].

A diagnostic dilemma may arise in the case of a single and isolated micro-endometrioma (< 10 mm) or ovarian endometriotic implant (≤ 5 mm), in which differentiation with an involuting functional cyst on MRI is limited, and its visualization or characterization on ultrasound almost impossible. The use of gadolinium injection could be limited as peripheral ring enhancement may not be visible in small hemorrhagic cysts due to their small size or resolution. Therefore, uncertain diagnosis should be considered to avoid systematic false-positive diagnosis of endometriosis. Depending on the level of clinical suspicion and available treatment options, follow-up with MRI may aid in obtaining a definitive diagnosis.

**Fig. 2:**
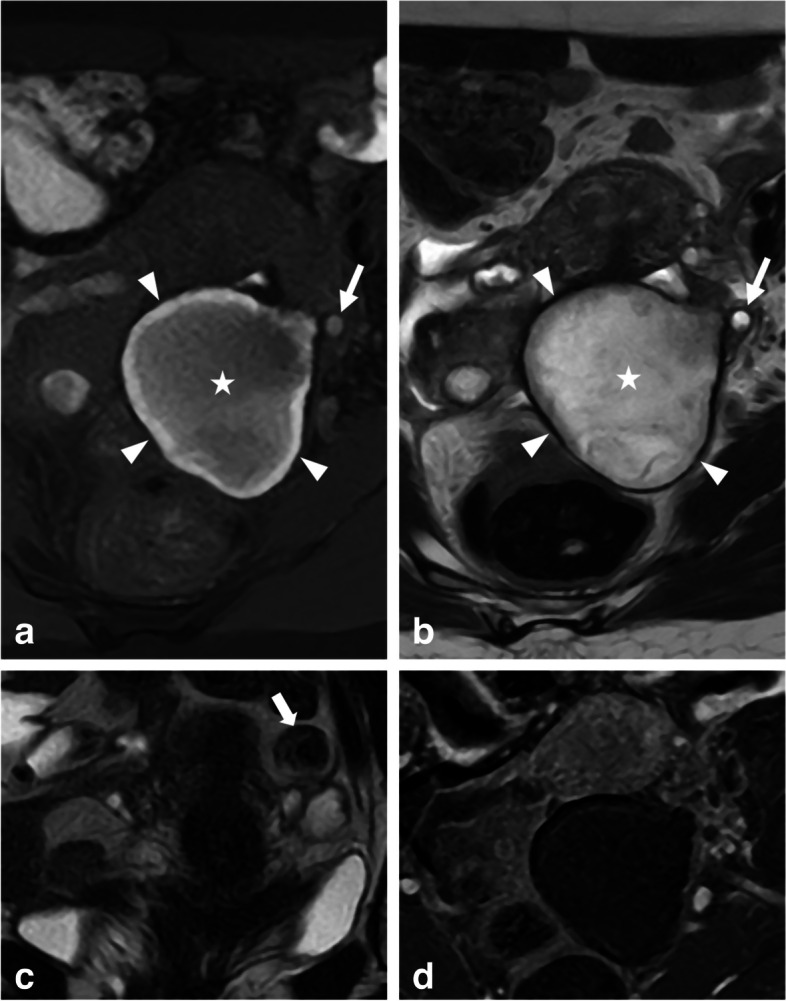
Ovarian severe ischemic necrosis, explored 1 month after presumed acute pelvic infection, in a 47-year-old woman. **a** Axial T1-W fat-suppressed MR image shows a T1-hyperintense left ovary (star) with a peripheral hyperintense rim (arrowheads), consistent with hemorrhage. A hemorrhagic follicle of the external cortex of the left ovary is identified (arrow). **b** Axial T2-W MR image shows enlarged centrally migrated left ovary (star) with peripheral hypointense rim (arrowheads). **c** Coronal T2-W MR image shows a pedicle twist (arrow). **d** Axial T1-W contrast-enhanced subtracted MR image shows complete absence of enhancement in the left ischemic ovary

Another pitfall is the presence of bilateral hemorrhagic cysts after controlled ovarian stimulation and follicular puncture, as many patients undergoing in vitro fertilization procedures are diagnosed with pelvic endometriosis, including endometriomas [26]. In this scenario, the clinical context and surgical report specifying the side of and the timeframe since the surgical puncture are relevant for accurate diagnosis. Post-procedure hematomas usually present as bilateral and multiple hemorrhagic ovarian cysts, with moderate to high signal intensity on T1-WI, generally not exceeding 1 cm in diameter (Supplemental - Figure 2). Distinguishing between prior bilateral micro-endometriomas and post-procedure hematomas can be challenging. Depending on clinical impact, follow-up MRI performed after 8–12 weeks remains the optimal approach, as complete or partial resolution of hemorrhagic cysts can assist in the differential diagnosis.

##### Ovarian ischemic necrosis

Adnexal torsion is defined by the twisting of the ovary and fallopian tube, causing vascular compromise and tissue damage, with varying degrees of ischemia, hemorrhagic infarction, and/or necrosis. It is commonly associated with a benign lesion in 80% of cases [27], but spontaneous torsion can occur without a predisposing mass, resembling endometriomas. The clinical manifestation of adnexal torsion can be variable, with acute lower abdominal pain associated with nausea, and vomiting. Chronic pelvic pain due to an unnoticed permanent torsion (Fig. 2) may also occur and be misleading. In this context, MRI is less often the first-line imaging technique since adnexal torsion diagnosis is rarely problematic, as the condition is usually evident based on clinical and other imaging findings such as massive ovarian enlargement, thickened twisted pedicle, hemorrhage in the stroma, abnormal displacement of the involved ovary and the uterus, and pelvic ascites [28]. Severe ischemic necrosis of the ovary may result in minimal or absent enhancement. However, the presence of T1-hyperintense signal after fat suppression is predictive of hemorrhagic infarction, which typically appears as a T1-hyperintense rim and may appear similar to a hemorrhagic cyst. Lastly, in rare cases of isolated tubal torsion, a thickened and dilated T1-hyperintense fallopian tube may show hemorrhage and lack of post-contrast enhancement [29]. It is worth highlighting that the O-RADS MRI score may not be applicable in certain acute scenarios where the signal is modified independently of the nature of the mass, like cases involving adnexal torsion or ectopic pregnancy.

##### Ectopic pregnancy

**Fig. 3:**
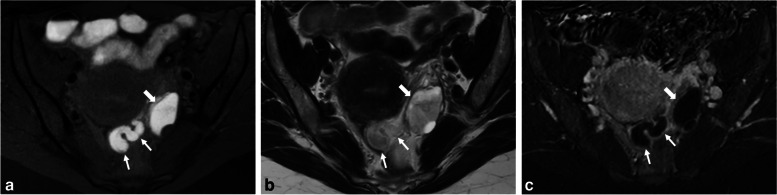
Ectopic tubal pregnancy with early tubo-ovarian abortion in a 39-year-old woman with subacute abdominal pain, and no medical history. **a** Axial T1-W fat-suppressed and (**b**) axial T2-W MR images show a left hemorrhagic cyst (thick arrows) corresponding to a hemorrhagic corpus luteum cyst of pregnancy, and an associated hematosalpinx (thin arrows). **c** Axial T1-weighted fat-suppressed contrast-enhanced subtracted MR image shows a significant enhancement of the fallopian tube wall (thin arrows), as well as the hemorrhagic corpus luteum cyst (thick arrow). Tubal pregnancy was confirmed after MRI exam with a positive HCG blood test

Tubal ectopic pregnancy (with or without a viable gestational sac) can cause nonspecific subacute pelvic pain, leading to diagnostic challenges if a pregnancy test is not performed prior to MRI. Differentiating between endometriosis with a single hematosalpinx presentation and ectopic pregnancy is important, given that tubal ectopic pregnancies frequently appear as a unilateral hematosalpinx (Fig. 3) [30]. A saclike cystic tubal structure with a thick wall may be absent; however, its presence should be checked [31]. Hemoperitoneum suggests rupture, supporting the possibility of an ectopic pregnancy. A T2-hyperintense solid component indicates the presence of trophoblastic tissue in ectopic pregnancy, aiding in differential diagnosis. Contrast-enhanced T1-WI can help detect the gestational sac and a strong wall enhancement encountered in tubal ectopic pregnancy. Of note, caution should be exercised when administering contrast agents if pregnancy is suspected. The absence of other MRI pelvic endometriotic features should also be in favor of an ectopic pregnancy. However, a recent metanalysis of 15 studies found that endometriosis is a possible risk factor for ectopic pregnancies, and therefore could be present in the case of an ectopic pregnancy [32]. In these cases, clinical context and a beta-HCG blood test should be performed to aid in the diagnosis.

#### Accessory cavitated uterine mass

Accessory cavitated uterine mass (ACUM) is a rare but underdiagnosed condition, mostly seen in young patients (< 30 years) presenting severe dysmenorrhea and chronic pelvic pain [33]. This congenital anomaly of the female genital tract, most likely due to gubernaculum dysfunction, consists of an isolated cavitated round mass, situated in close proximity to the insertion point of the uterine round ligament, and lined by a functional endometrium [34]. ACUM could be defined on MRI as a functioning and non-communicating accessory horn located in the external myometrium or the broad ligament of an otherwise normal uterus [35]. The broad ligament presentation is less common but can be misdiagnosed as an endometrioma when it is close to the ovary. ACUM differs from endometrioma by its extra-ovarian location, and its specific MRI features, including a central round cavitated mass filled with hematometra (high signal intensity on T1-WI and shading effect or fluid-fluid level on T2-WI), and a well-defined thick T1- and T2-hypointense peripheral ring of muscular tissue resembling the junctional zone (inner myometrium) with low enhancement if contrasts agents are used (Supplemental - Figure 3) [35]. Laparoscopic surgery is the primary option, aiming to preserve fertility and relieve symptoms, allowing for histological diagnosis [35]. Hormone-suppressive therapy may also be considered, often leading to the resolution of central hemorrhagic hypersignal on follow-up MRI.

### Hyperproteic T1-hyperintense cyst

Protein-containing lesions may exhibit a T1-hyperintense signal on fat-suppressed T1-WI, depending on the amount of free water and viscosity within the lesion, resulting in shortening of T1 relaxation times [36]. The T2 signal is typically sensitive to variations in tissue water content but may be less sensitive and require significant dehydration for the signal to decrease [37]. Small lesions with protein content tend to dehydrate faster, resulting in an increase in their T1 signal and a relative decrease in their T2 signal.

#### Adnexal lesions

##### Chronic pelvic inflammatory disease

**Fig. 4:**
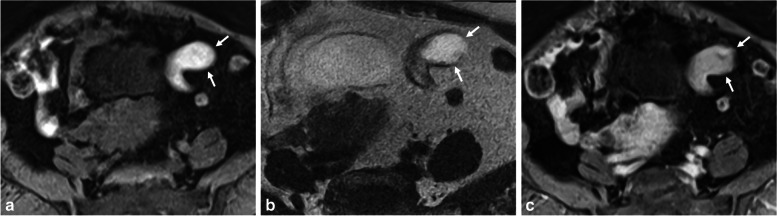
Chronic hydrosalpinx in a 58-year-old woman with pelvic pain and history of PID. **a** Axial T1-W fat-suppressed, (**b**) axial T2-W, and (**c**) axial T1-W fat-suppressed contrast-enhanced MR images show a fluid-filled fallopian tube (arrows) with high T1- and T2-signal intensity. Note the absence of fat stranding, peritonitis, or significant adnexal enhancement

Pelvic inflammatory disease is the most common cause of tubal occlusion and hydrosalpinx [38]. It is one of the most frequent gynecologic causes of emergency department visits. Pelvic inflammatory disease is usually caused by an infection ascending from the lower genital tract [39]. In acute cases, an MRI is not necessary for symptomatic women. However, the symptoms are often mild and nonspecific (fever and increased white blood cell count may be absent) [40], making it challenging for clinicians to reach the correct diagnosis, particularly in chronic cases. In such situations, a hydrosalpinx develops as a result of adhesions and scarring, and it may appear as a dilated fallopian tube folding upon itself, forming a sausage-like C- or S-shaped cystic mass [41]. The signal intensity of the fluid in the fallopian tube depends on the cause of the blockage [42]. The tubal content in chronic salpingitis can exhibit a T1-hyperintense signal due to the retention of mucus secreted by the mucosal epithelium (Fig. 4), rated O-RADS MRI 3. It is often unilateral, isolated, and lacks other suggestive signs of pelvic endometriosis. Contrast-enhanced T1-WI can also aid in the differential diagnosis of subacute episodes by demonstrating either no enhancement or low enhancement in cases of chronic infection (excluding concurrent infectious episodes).

**Fig. 5:**
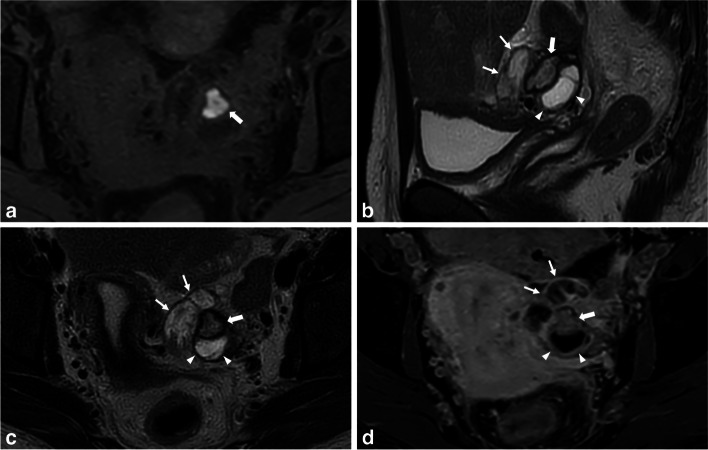
Subacute tubo-ovarian abscess in a 30-year-old woman with subacute pelvic pain (3 weeks ago) and persistent left pelvic pain. **a** Axial T1-W fat-suppressed MR image shows a T1-hyperintense cystic ovarian lesion (thick arrow), corresponding to proteic and hemorrhagic content. **b** Sagittal and (**c**) axial T2-W MR images show an ovarian well-defined cystic cavity with intermediate T2-signal intensity, surrounded by a hypointense peripheral rim (thick arrows). Note the presence of an adjacent hydrosalpinx with heterogeneous signal intensity (thin arrows). Of note, the presence of a homolateral T2-hyperintense functional ovarian cyst (arrowheads). **d** Axial T1-W fat-suppressed contrast-enhanced MR image shows enhancement of the collapsed walls of the remnant ovarian abscess (thick arrow) as well as the walls of the hydrosalpinx (thin arrows), with obliteration of the fat planes around the left ovary. Of note, the presence of a homolateral functional ovarian cyst showing a peripheral enhancement (arrowheads)

Less commonly, a persistent remnant ovarian abscess can be observed in subacute or chronic tubo-ovarian presentations [43]. The signal intensity of the abscess can vary depending on its viscosity or protein concentration, displaying T1-hyperintense content and high signal intensity on T2-WI (Fig. 5) and DWI. A DWI-hyperintense rim along the inner layer of the thickened wall indicates granulation tissue with microscopic bleeding, accompanied by moderate to sustained post-contrast enhancement [29]. In contrast, endometriomas do not exhibit post-contrast enhancement and typically display homogeneous high signal intensity on DWI [25]. It is important to note that both endometriomas and the abscess cavity may exhibit restricted diffusion, but the signal heterogeneity on DWI is suggestive of an abscess process.

Tubo-ovarian infection must be distinguished from superinfection of an endometrioma, a rare condition occurring in around 2% of cases [44]. Fever is present in only 30% of cases, and about 60% show signs of biological inflammatory response [45]. Endometriomas create a conducive environment for bacterial growth due to aged blood (acting as an effective culture medium for bacterial growth) [46], increasing the risk of infection. Infections can occur through bacterial ascent from the vaginal orifice to the fallopian tube, direct inoculation (e.g., during hysterosalpingography), or digestive translocation, especially in cases involving the rectosigmoid. Superinfection primarily affects the endometrioma rather than the fallopian tube, distinguishing it from typical tubo-ovarian infections. Infected endometriomas tend to be large and may exhibit changes in signal intensity on MRI, such as loss of high signal intensity on T1-WI and/or loss of shading on T2-WI due to liquefaction. Contrast agents do not aid in distinguishing between these entities, as both show significant wall enhancement with adjacent fatty infiltration in intermediate signal intensity on T2-WI. In such cases, the diagnosis is often based on monitoring the infectious episode, particularly when other suggestive pelvic endometriosis lesions are absent.

##### Epithelial cystic tumors

Serous cystadenomas are the most common benign epithelial neoplasms in women of reproductive age [47], and overall, in ovarian neoplasms [48]. Unilateral lesions are more common, but bilateral cases can occur in up to 20% of cases [49]. They typically appear as fluid-filled cystic lesions that are T2-hyperintense and T1-hypointense, rated O-RADS MRI 2. In rare cases, they may show moderate to high signal intensity on T1-WI due to proteinaceous secretions, cell desquamation, and microbleeding, and should suggest a serous borderline cystadenoma [50]. However, the signal intensity on T1-WI remains lower than that of blood, and the shading effect on T2-WI is usually absent. Moreover, suspicion of malignancy should be raised by the presence of enhancing solid (nonfatty, nonfibrous) components, thickened irregular septa (> 3 mm), papillary projections, or a mural nodule, encountered in borderline or invasive tumors, rated O-RADS MRI 4 or 5 [48]. Differentiating a T1-hyperintense malignant epithelial cystic tumor from a malignant transformation of an endometrioma can indeed be challenging. Malignant transformations of endometriomas are relatively rare, occurring in approximately 1–2% of cases [51]. These transformations involve the development of malignancies within pre-existing endometriomas. In the context of imaging, several features can help differentiate between the two conditions. Signs suggestive of malignant transformation of an endometrioma include an increase in size, presence of solid tissue, loss of shading (seen as an increased signal on T2-WI), and wall enhancement [52]. Lastly, borderline seromucinous cystadenomas should be considered in the differential diagnosis of malignant transformations of endometriomas. Such ovarian borderline tumors are relatively uncommon but not rare neoplasms, and they can involve both ovaries in up to 40% of patients [53], often being associated with endometriosis in 30–50% of cases [54]. On imaging, a key finding of borderline seromucinous cystadenomas is the presence of T2-hyperintense papillary projections, reflecting their mucinous nature. In contrast, malignant endometriomas typically exhibit contrast-enhanced mural nodules instead [55].

##### Paratubal cysts

**Fig. 6:**
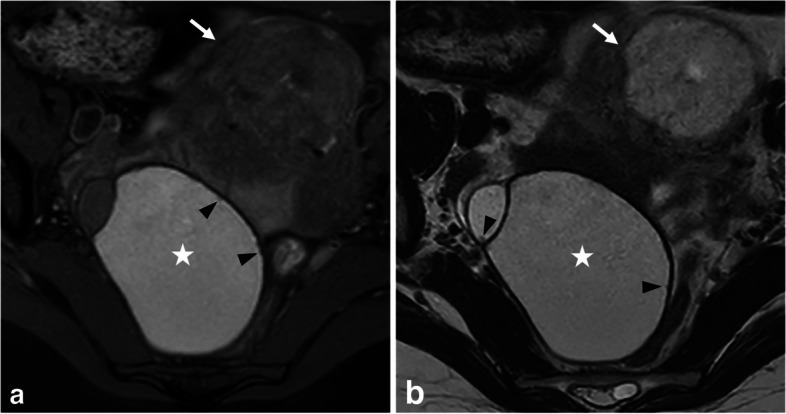
Right-sided paratubal serous papillary cystadenofibroma in a 29-year-old woman with right pelvic pain during first trimester pregnancy. **a** Axial T1-W fat-suppressed and (**b**) axial T2-W MR images show a bilocular paratubal cystic lesion (star) with a T1- and T2-hyperintense posterior loculus (star). The wall is irregular with endocystic tiny papillary projections (arrowheads). Note the gravid uterus (arrows)

Paratubal cysts, also known as para-ovarian cysts, originate from vestigial tissue of the Wolffian mesonephric ducts [56]. They are located in the broad ligament at the fimbriae end of the fallopian tube and account for approximately 10% of all adnexal masses [57]. On MRI, paratubal cysts appear as well-defined, round cystic structures that are separate from the ovary (negative beak sign) and located on the surface of the tube, rated O-RADS MRI 2. It is important to check for adjacent or contralateral paratubal cysts, as bilateral cysts are frequently observed. Occasionally, they may exhibit T1-hyperintense content due to viscosity or high protein content but do not show a shading effect on T2-WI (Fig. 6).

#### Vagina, vulva, and urethra

A vaginal cyst may be secondary to an embryologic derivative, ectopic tissue, or represent a urologic abnormality; vulvar cysts are most often related to dilated glands. Epithelial inclusion cyst, Gartner’s duct cyst, and Bartholin’s gland cyst represent common vulvovaginal cystic abnormalities not to be confused with endometriosis. It is important to note that endometriosis in this region is exceptionally rare, especially in the absence of prior vaginal surgeries or a history of childbirth complications.

##### Gartner’s duct cysts

Gartner’s duct cysts are mesonephric remnants within the vagina. They are often asymptomatic, especially in the absence of complications, but large cysts can cause urinary symptoms by pressing on the urethra [58]. They are usually located in the anterolateral vaginal wall but can be mistaken for cystic vaginal endometriosis when found in the posterosuperior wall [59]. High signal intensity on T1-WI is typically caused by high proteinaceous content, resulting in heterogeneous signal intensity. Additionally, there may be a potential for low signal intensity on T2-WI (Supplemental - Figure 4). Gartner’s duct cysts are usually single, more rarely multiple, and may be associated with renal abnormalities such as agenesis, dysplasia, or cross ectopy [60]. Adding T2 large-field-of-view sequences is helpful in this context to complete imaging evaluation.

##### Epithelial inclusion cysts

**Fig. 7:**
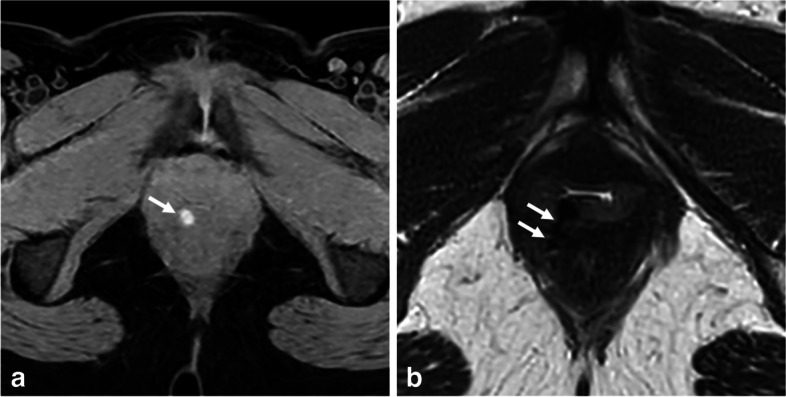
Vaginal epithelial inclusion cyst in a 35-year-old woman with non-cyclic perineal pain and palpable vaginal induration, 1 year after childbirth with episiotomy. **a** Axial T1-W fat-suppressed MR image shows a single small T1-hyperintense cyst (arrow) in the posterior vaginal wall. **b** Axial T2-W MR image shows a T2-hypointense cyst and T2-hypointense thin linear fibrous scar tissue within the puborectalis muscle (arrows), consistent with an episiotomy scar

Epithelial inclusion cysts are the most prevalent type of vaginal cysts, comprising approximately 23% of all cases [60]. These cysts occur when epithelial tissue becomes focally trapped due to trauma or prior surgeries like episiotomy. They are commonly found on the lower posterior vaginal wall or the vaginal cuff (Fig. 7). Distinguishing between a typical scar and an endometriotic episiotomy scar involves examining the patient’s surgical history, such as hysterectomy or episiotomy (either mediolateral or midline), assessing the nature of perineal pain (whether it is cyclic or non-cyclic), and conducting thorough physical examinations. Furthermore, specific imaging features, like differentiating between single or very few versus multiple T1-hyperintense hemorrhagic microcysts and thin linear versus thick fibromuscular T2-hypointense scars enclosing hemorrhagic implants (Fig. 7), offer valuable clues, preventing premature assumptions of an endometriotic scar.

##### Bartholin’s gland cysts

Bartholin’s glands, or greater vestibular glands, arise in the superficial perineal pouch of the urogenital triangle. When the ducts of these glands become obstructed, it leads to retention of secretions and the formation of cysts [61]. Bartholin’s gland cysts are the most common type of vulvar cysts, mostly ranging in size from 1 to 4 cm. While many patients with Bartholin’s gland cysts are asymptomatic, some may experience mild dyspareunia during vaginal penetration, which needs to be differentiated from deep dyspareunia associated with endometriosis. It is important to note that the specific anatomical location of these cysts on the posterolateral surface of the vestibule, often occurring bilaterally, should not be mistaken for cystic endometriotic implants in patients with a history of vaginal delivery (with or without episiotomy) or prior transvaginal surgeries. On imaging, Bartholin’s gland cysts with high proteinaceous content exhibit high signal intensity on T1-WI, along with heterogeneous and low signal intensity on T2-WI (Supplemental - Figure 5).

##### Skene’s gland cysts

Skene’s glands, or periurethral glands, are tubule-alveolar structures located at the 3 and 9 o’clock positions along the distal two-thirds of the urethra, providing lubrication [62]. These are typically round and unilocular fluid-filled cysts. Homogeneous T1-hyperintensity, suggesting high proteinaceous content, can help in their detection and physical examination identification [61] (Supplemental - Figure 6). It is essential to differentiate these cysts from urethral diverticulum considering a mutlicystic or circular appearance, the presence of a connection with the urethra (Supplemental - Figure 7) and the patient’s medical history.

#### Peritoneum

##### Multicystic peritoneal mesothelioma

**Fig. 8:**
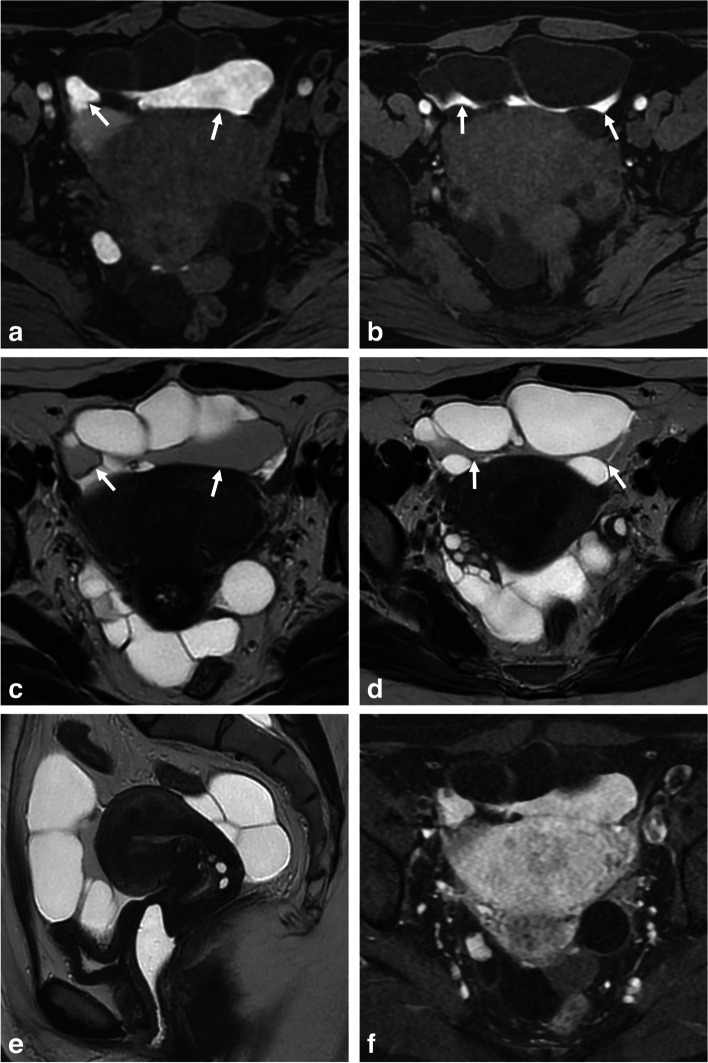
Multicystic peritoneal mesothelioma in a 38-year-old woman with chronic pelvic pain addressed for suspicion of endometriosis. **a, b** Axial T1-W fat-suppressed MR images show an anterior and posterior pelvic multicystic lesion with some loculi showing hyperintense content (arrows). **c, d** Axial and (**e**) sagittal T2-W MR images show a thin-walled multiple grape-like clusters lesion, with intermediate signal intensity in thick-content loculi (arrows), gently molding to the polymyomatous uterus and normal ovaries, as well as the pelvic wall. **f** Axial fat-suppressed contrast-enhanced subtracted T1-W image shows no thick or irregular wall enhancement of the multicytic lesion, nor tissular portion

Multicystic peritoneal mesothelioma is a rare benign neoplasm arising from the peritoneum [63]. It is often found in women of reproductive age, with a preferential location in the pelvis, and can be discovered incidentally or present with symptoms such as heaviness, distension, or pain [64]. These mesotheliomas appear as well-defined cystic lesions, typically over 10 cm in size, with grape-like clusters and possible mild septal enhancement. In some cases, the cystic nature of the lesions and debris within them may cause a mildly elevated signal on T1-WI (Fig. 8). This can occasionally lead to confusion with endometriotic lesions, particularly in cases of severe peritoneal involvement with combined cystic peritoneal endometriotic lesions and pelvic adhesions with peritoneal hemorrhagic inclusion cysts. However, the absence of deep endometriotic lesions and normal ovaries helps differentiate multicystic peritoneal mesothelioma. In addition to the grape-like appearance which argues for a full-fledged lesion (versus adhesions), the presence of multi-focal peritoneal involvement further supports the diagnosis. Therefore, a dedicated peritoneal MRI in addition to pelvic MRI is necessary [65]. Patients suspected of having multicystic peritoneal mesothelioma should be referred to a specialized center for a comprehensive evaluation, which includes obtaining an accurate pathological diagnosis. Treatment options, such as complete cytoreduction with or without hyperthermic intraperitoneal chemotherapy, can be discussed to minimize recurrence or potential malignant transformation [64].

#### Retrorectal space

Retrorectal cystic lesions in adults are rare and most cases are congenital. Developmental cysts are the most common congenital entities found in the retrorectal space and include tailgut cysts (also known as retrorectal hamartomas), epidermoid cysts, and dermoid cysts [66].

##### Tailgut cysts

**Fig. 9:**
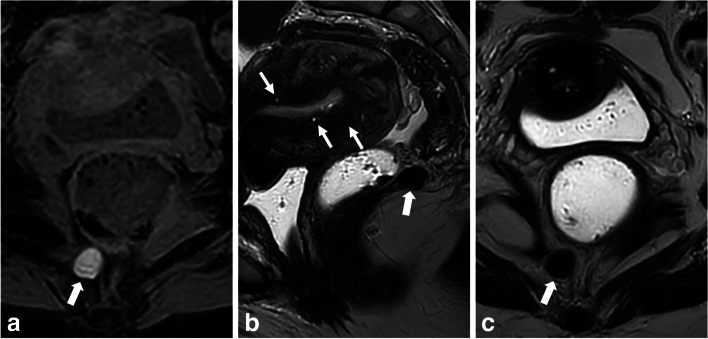
Retrorectal hamartoma (tailgut cyst) in a 46-year-old patient with gradually worsening dysmenorrhea and menometrorrhagia. **a** Axial fat-suppressed T1-W MR image shows a small-sized, well-defined and thin-walled T1-hyperintense cyst (thick arrow) isolated in the retrorectal space, along the right side of the anococcygeal raphe. **b** Sagittal and (**c**) axial T2-W MR images show a distinct T2-hypointense retrorectal cyst (thick arrows). Note the absence of posterior deep infiltrating endometriosis lesions but the presence of internal adenomyosis (thin arrows) consistent with the symptomatology

Tailgut cysts are rare congenital cysts thought to arise from the embryonic postanal gut tissue [67]. They are the most common asymptomatic retrorectal developmental cysts, often incidentally found in adult women [68]. Despite not aligning with the pathogenic theories of endometriosis dissemination, tailgut cysts may routinely be misdiagnosed as cystic endometriosis lesions. Indeed, while deep extra or retroperitoneal endometriosis mainly consist of an extension from DIE, tailgut cysts appear on MRI as isolated thin-walled uni- or multilocular cystic masses [69], with low signal intensity on T1-WI, although they can be T1-hyperintense depending on their mucoid content, and exhibit variable signal intensity on T2-WI ranging from T2-hyperintense to T2-hypointense (Fig. 9) [70]. Contrast-enhanced T1-WI can reveal thick and regular septa and vegetations, which are indicative of possible malignant transformation to adenocarcinoma [71]. Despite often being discovered incidentally, recent retrospective evidence highlights that surgical intervention, accompanied by preoperative MRI, is the recommended approach for cystic and solid retrorectal tumors due to the potential risk of malignant transformation, even in incidental cases, and ensures a definitive histological diagnosis [72].

##### Other retrorectal cysts

Other retrorectal developmental cysts, such as teratomas and epidermoid cysts, are even rarer [66]. Teratomas are uniloculated cysts that contain fat tissue and other components, while epidermoid cysts are large cysts with heterogeneous dense content. These cysts are less likely to mimic endometriotic cysts.

#### Urachus

##### Urachal cysts

The urachus is a ductal remnant that forms during early embryological development, connecting the bladder dome to the umbilicus [73]. Normally, the urachus undergoes involution and its lumen is obliterated. However, in some cases, remnants of the urachus can persist and result in various abnormalities such as urachal fistula, umbilical-urachal sinus, urachal cyst, and vesico-urachal diverticulum [74]. These abnormalities are often discovered incidentally during MR imaging performed for unrelated reasons. Urachal cysts occur when a segment of the urachus fails to close, most commonly in the lower third of the urachal tract. These cysts are typically small and asymptomatic. On imaging, the presence of a fluid-filled structure along the midline of the urachal course indicates an uncomplicated cyst. The signal intensity within the cyst can vary, ranging from low to high signal intensity on T1-WI depending on the presence of mucoid material and proteinaceous content (Supplemental - Figure 8).

### Artifacts

Having a thorough understanding of MRI artifacts is crucial when conducting pelvic imaging. Their presence can mask, distort, or mimic pathological lesions, potentially leading to misinterpretation of the images.

#### Vascular artifacts

##### Flow-related enhancement

**Fig. 10:**
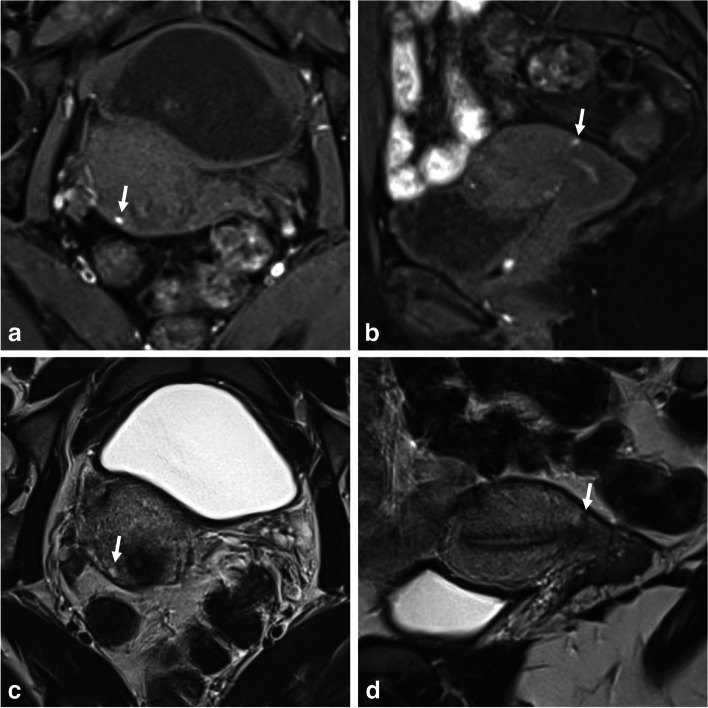
Flow-related enhancement artifact, also known as “the entry slice phenomenon”, in a 36-year-old woman with chronic pelvis pain and dysmenorrhea. **a** Axial and (**b**) sagittal reconstruction T1-W fat-suppressed MR image show a bright spot (arrows) in close proximity with the right USL insertion, also present on contiguous slices (not shown). **c** Axial and (**d**) sagittal T2-W MR images show a sub-serous vein (arrows) in place of the T1 bright spot, near the right USL insertion

Flow-related enhancement artifact, also known as the “entry slice phenomenon,” refers to the bright signal of blood within the initial slices of an imaging acquisition. It is commonly seen with gradient-echo sequences and, to a lesser extent, spin-echo sequences [75]. This artifact occurs because flowing blood entering a slice is unsaturated, leading to increased signal on MR images [75]. To address this artifact, spatial saturation bands can be used to remove the undesired signal enhancement. However, in the pelvic region, arteries and large-caliber fast-flowing veins oriented in the phase-encoding direction may still show increased signal intensity on T1-WI, resulting in paradoxical enhancement (Fig. 10). It is crucial to distinguish this artifact from endometriotic implants, especially in the parametrium or adnexal region (Supplemental - Figure 9). Factors such as anatomical location, linear or serpiginous appearance, presence of flow-voids on T2-WI, and gadolinium-enhanced sequences can help confirm suspicions of vascular origin. The artifact typically spans multiple consecutive slices but fades with distance. In challenging cases, inversion recovery sequences or fat-saturated black-blood double inversion recovery sequences can aid in detecting and differentiating this artifact from true pathology.

##### Vascular ghosts

Vascular ghost artifacts in the pelvis result from phase variations caused by pulsatile arterial flow, typically originating from the iliac vessels [75]. Pulsatile flow can lead to ghosting in the phase-encoding direction, resulting in one or more T1-hyperintense ghost images. Vascular ghost artifacts can overlap with gynecologic structures, particularly the ovaries, which are situated in the path of the iliac vessels (Supplemental - Figure 10) [76]. The repeating pattern of this artifact in the phase direction, along with the absence of corresponding findings on other sequences, usually facilitates its identification. However, in challenging cases, additional sequences with a reversal of the phase-encoding direction can be used to help rule out this artifact.

#### Calcification artifacts

**Fig. 11:**
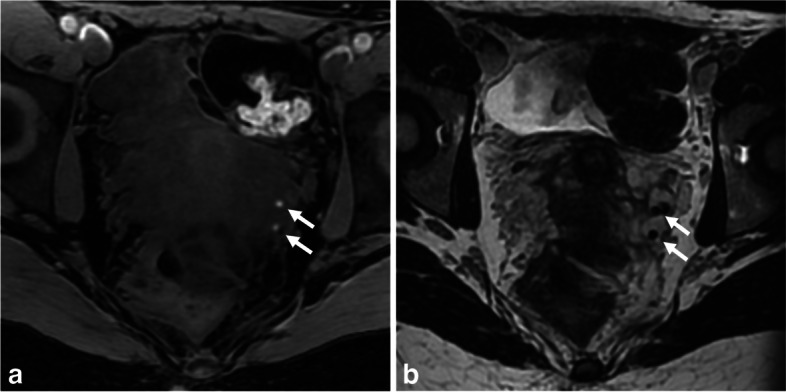
Parametrial left-sided phlebolith in an 18-year-old woman. **a** Axial T1-W and (**b**) axial T2-W MR images show a punctiform T1-hyperintense “spot” matching a T2 “dark spot” within the lumen of a parametrial vein (arrows)

In specific circumstances, calcium can appear T1-hyperintense, despite being a diamagnetic substance [77]. The T1 signal intensity increases until a certain calcium concentration is reached (30% by weight), primarily due to T1 shortening. However, for high calcium concentrations, the T1-weighted signal intensity decreases due to the dominant effect of low proton density. Nevertheless, an increase in the surface area of calcifications leads to an elevated T1-weighted relaxivity [76]. Phleboliths, which are calcium concretions found within the endoluminal vessels, can exhibit a bright T1-hyperintense signal after fat suppression, especially in the parametrium veins of the pelvic cavity. Their round shape, endovascular location, and T2-hypointense appearance help in their recognition (Fig. 11). Computed tomography scan, if already performed for another reason and available at the time of diagnosis, can also assist in identifying phleboliths.

#### Lack of fat suppression

Fat suppression techniques are commonly used in MR imaging to either detect or suppress the signal from adipose tissue. Three main methods are used: fat saturation, inversion recovery, and opposed-phase imaging, each with their own advantages, disadvantages, and potential pitfalls [78]. Short Inversion Time Inversion Recovery (STIR) sequences, frequently used in musculoskeletal MR imaging, may not completely suppress the signal from tumors containing fat. This is due to variations in T1 relaxation time between tissues containing fat and pure fat itself. Consequently, failure in fat suppression can lead to misleading high signal intensity, making it challenging to differentiate between endometriomas and mature cystic teratomas, or, less commonly, sequelae of epiploic appendagitis. Opposed-phase imaging relies on the fact that the lipid signal and water signal are additive on in-phase images, but on opposed-phase images, the signal represents the difference between the lipid and water signals. As a result, opposed-phase imaging helps to reduce the signal from fatty tissue. The Dixon method acquires both in-phase and opposed-phase images, enabling the production of pure water and pure lipid images, which can aid in the differential diagnosis process.

### Feces

**Fig. 12:**
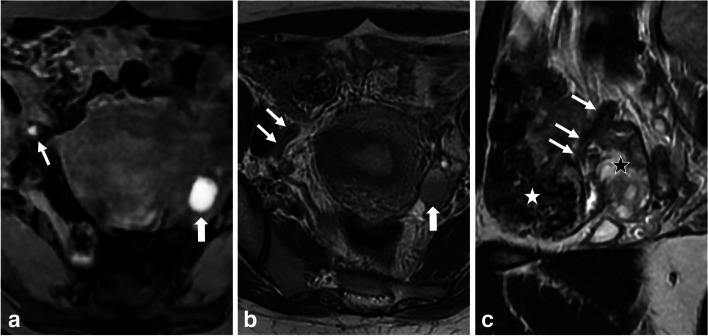
Normal appendix filled with feces in a 34-year-old woman with chronic pelvic pain and medical history of endometriosis referred for infertility issues. **a** Axial T1-W fat-suppressed MR image shows a T1-hyperintense “spot” in the right lateral pelvic side (thick arrow). **b** Axial and (**c**) sagittal T2-W MR images show that the T1-hyperintense “spot” is located in a tubular structure (thin arrows) referring to the appendix running over the right ovary (black star), consistent with endoluminal feces concretion (white star). Note the presence of a left endometrioma with T2 shading (thick arrows in **a** and **b**)

Proteinaceous concretions or fecal matter within the intestinal lumen exhibit increased signal on T1-WI [79] and should not be mistaken for endometriosis, especially when dealing with coproliths (Supplemental - Figure 11) or appendicoliths in the pelvic area. Confirmation of an appendicolith can be achieved through multiplanar reconstruction on 3D T2-WI, along with the presence of an intraluminal increased signal on T1-WI (Fig. 12). Endometriosis of the appendix typically involves the outer wall layers with extrinsic involvement, resulting in stenosis and possible appendicular distension, also known as mucocele.

### Melanin

Vulvar or vaginal melanomas are very rare tissular malignancies that exhibit T1-hyperintense signal intensity, attributed to the paramagnetic properties of melanin and methemoglobin, with corresponding low to intermediate signal intensity on T2-WI [80]. Contrast-enhanced subtracted MR images can be helpful in confirming the presence of tissue malignancies (Supplemental - Figure 12).

## Conclusion

In summary, many pitfalls and differential diagnoses should be known to the radiologist specializing in pelvic endometriosis. T1-hyperintense signal abnormalities may mimic lesions and condition-like mimickers that are radiologically similar to endometriosis, but different in every respect. Acknowledging key MR findings for the differential diagnosis of endometriosis is therefore essential in the diagnostic accuracy and proper treatment approach for such entities to improve clinical and surgical workflows.

### Supplementary Information

**Additional file 1.**

## Data Availability

The data of cases in the manuscript are available from the corresponding author on reasonable request.

## Abbreviations

ACUM: Accessory and cavitated uterine mass
DIE: Deep infiltrative endometriosis
DWI: Diffusion-weighted images
FS T1-WI: Fat-suppressed T1-weighted images
MRI: Magnetic resonance imaging
O-RADS MRI: Ovarian-Adnexal Reporting and Data System on Magnetic Resonance Imaging
T1 T2-WI: T1 T2-weighted images
USL: Uterosacral ligament

## References

[1] Taylor HS, Kotlyar AM, Flores VA (2021). Endometriosis is a chronic systemic disease: clinical challenges and novel innovations. Lancet.

[2] Chapron C, Marcellin L, Borghese B, Santulli P (2019). Rethinking mechanisms, diagnosis and management of endometriosis. Nat Rev Endocrinol.

[3] Tomassetti C, Johnson NP, International working group of AAGL, ESGE, ESHRE and WES (2021). An International Terminology for Endometriosis, 2021. J Minim Invasive Gynecol.

[4] Audebert A, Petousis S, Margioula-Siarkou C (2018). Anatomic distribution of endometriosis: A reappraisal based on series of 1101 patients. Eur J Obstet Gynecol Reprod Biol.

[5] Simoens S, Dunselman G, Dirksen C (2012). The burden of endometriosis: costs and quality of life of women with endometriosis and treated in referral centres. Hum Reprod.

[6] Bazot M, Daraï E (2017). Diagnosis of deep endometriosis: clinical examination, ultrasonography, magnetic resonance imaging, and other techniques. Fertil Steril.

[7] Rousset P, Florin M, Bharwani N (2023). Deep pelvic infiltrating endometriosis: MRI consensus lexicon and compartment-based approach from the ENDOVALIRM group. Diagn Interv Imaging.

[8] Nisenblat V, Bossuyt PMM, Farquhar C (2016). Imaging modalities for the non-invasive diagnosis of endometriosis. Cochrane Database Syst Rev.

[9] Medeiros LR, Rosa MI, Silva BR (2015). Accuracy of magnetic resonance in deeply infiltrating endometriosis: a systematic review and meta-analysis. Arch Gynecol Obstet.

[10] Bazot M, Bharwani N, Huchon C (2017). European society of urogenital radiology (ESUR) guidelines: MR imaging of pelvic endometriosis. Eur Radiol.

[11] Togashi K, Nishimura K, Kimura I (1991). Endometrial cysts: diagnosis with MR imaging. Radiology.

[12] Defrère S, Lousse JC, González-Ramos R (2008). Potential involvement of iron in the pathogenesis of peritoneal endometriosis. Mol Hum Reprod.

[13] Corwin MT, Gerscovich EO, Lamba R (2014). Differentiation of Ovarian Endometriomas from Hemorrhagic Cysts at MR Imaging: Utility of the T2 Dark Spot Sign. Radiology.

[14] Jeong Y-Y, Outwater EK, Kang HK (2000). Imaging Evaluation of Ovarian Masses. Radiographics.

[15] McDermott S, Oei TN, Iyer VR, Lee SI (2012). MR Imaging of Malignancies Arising in Endometriomas and Extraovarian Endometriosis. Radiographics.

[16] Sadowski EA, Thomassin-Naggara I, Rockall A (2022). O-RADS MRI Risk Stratification System: Guide for Assessing Adnexal Lesions from the ACR O-RADS Committee. Radiology.

[17] Thomassin-Naggara I, Poncelet E, Jalaguier-Coudray A (2020). Ovarian-Adnexal Reporting Data System Magnetic Resonance Imaging (O-RADS MRI) Score for Risk Stratification of Sonographically Indeterminate Adnexal Masses. JAMA Netw Open.

[18] Foti PV, Farina R, Palmucci S (2018). Endometriosis: clinical features, MR imaging findings and pathologic correlation. Insights Imaging.

[19] Coutinho A, Bittencourt LK, Pires CE (2011). MR Imaging in Deep Pelvic Endometriosis: A Pictorial Essay. Radiographics.

[20] Allen BC, Hosseinzadeh K, Qasem SA (2014). Practical Approach to MRI of Female Pelvic Masses. AJR Am J Roentgenol.

[21] Jha P, Sakala M, Chamie LP (2020). Endometriosis MRI lexicon: consensus statement from the society of abdominal radiology endometriosis disease-focused panel. Abdom Radiol (NY).

[22] Thomassin-Naggara I, Perrot N, Salem C, Bazot M (2007). Technique IRM de caractérisation des masses annexielles. Imag Femme.

[23] Busard MPH, Mijatovic V, van Kuijk C (2010). Magnetic resonance imaging in the evaluation of (deep infiltrating) endometriosis: The value of diffusion-weighted imaging. J Magn Reson Imaging.

[24] Lee NK, Kim S, Kim KH (2016). Diffusion-weighted magnetic resonance imaging in the differentiation of endometriomas from hemorrhagic cysts in the ovary. Acta Radiol.

[25] Khashper A, Addley HC, Abourokbah N (2012). T2-Hypointense Adnexal Lesions: An Imaging Algorithm. Radiographics.

[26] Garcia-Velasco JA, Somigliana E (2009). Management of endometriomas in women requiring IVF: to touch or not to touch. Hum Reprod.

[27] Varras M, Tsikini A, Polyzos D (2004). Uterine adnexal torsion: pathologic and gray-scale ultrasonographic findings. Clin Exp Obstet Gynecol.

[28] Béranger-Gibert S, Sakly H, Ballester M (2016). Diagnostic Value of MR Imaging in the Diagnosis of Adnexal Torsion. Radiology.

[29] Iraha Y, Okada M, Iraha R, Azama K CT and MR Imaging of Gynecologic Emergencies. Radiographics 37(5):1569–1586 10.1148/rg.2017160170 28753380

[30] Parker RA, Yano M, Tai AW (2012). MR Imaging Findings of Ectopic Pregnancy: A Pictorial Review. Radiographics.

[31] Tamai K, Koyama T, Togashi K (2007). MR features of ectopic pregnancy. Eur Radiol.

[32] Yong PJ, Matwani S, Brace C (2020). Endometriosis and Ectopic Pregnancy: A Meta-analysis. J Minim Invasive Gynecol.

[33] Acien P, Bataller A, Fernandez F (2012). New cases of accessory and cavitated uterine masses (ACUM): a significant cause of severe dysmenorrhea and recurrent pelvic pain in young women. Hum Reprod.

[34] Acién P, Acién M (2016). Diagnostic imaging and cataloguing of female genital malformations. Insights Imaging.

[35] Peyron N, Jacquemier E, Charlot M (2019). Accessory cavitated uterine mass: MRI features and surgical correlations of a rare but under-recognised entity. Eur Radiol.

[36] Hayashi Y, Tachibana O, Muramatsu N (1999). Rathke cleft cyst: MR and biomedical analysis of cyst content. J Comput Assist Tomogr.

[37] McCarthy S, Scott G, Majumdar S (1989). Uterine junctional zone: MR study of water content and relaxation properties. Radiology.

[38] Kim MY, Rha SE, Oh SN (2009). MR Imaging Findings of Hydrosalpinx: A Comprehensive Review. Radiographics.

[39] Soper DE (2010). Pelvic inflammatory disease. Obstet Gynecol.

[40] Revzin MV, Mathur M, Dave HB (2016). Pelvic Inflammatory Disease: Multimodality Imaging Approach with Clinical-Pathologic Correlation. Radiographics.

[41] Outwater EK, Siegelman ES, Chiowanich P (1998). Dilated fallopian tubes: MR imaging characteristics. Radiology.

[42] Foti PV, Ognibene N, Spadola S (2016). Non-neoplastic diseases of the fallopian tube: MR imaging with emphasis on diffusion-weighted imaging. Insights Imaging.

[43] Stenchever MA (2001). Comprehensive gynecology.

[44] Grammatikakis I, Evangelinakis N, Salamalekis G (2009). Prevalence of severe pelvic inflammatory disease and endometriotic ovarian cysts: a 7-year retrospective study. Clin Exp Obstet Gynecol.

[45] Landers DV, Sweet RL (1983). Tubo-ovarian abscess: contemporary approach to management. Rev Infect Dis.

[46] Chen M-J, Yang J-H, Yang Y-S, Ho H-N (2004). Increased occurrence of tubo-ovarian abscesses in women with stage III and IV endometriosis. Fertil Steril.

[47] Chang HC, Bhatt S, Dogra VS (2008). Pearls and Pitfalls in Diagnosis of Ovarian Torsion. Radiographics.

[48] Taylor EC, Irshaid L, Mathur M (2021). Multimodality Imaging Approach to Ovarian Neoplasms with Pathologic Correlation. Radiographics.

[49] Seidman JD, Mehrotra A (2005). Benign ovarian serous tumors: a re-evaluation and proposed reclassification of serous “cystadenomas” and “cystadenofibromas”. Gynecol Oncol.

[50] Nakai G, Yamada T, Yamamoto K (2018). MRI appearance of ovarian serous borderline tumors of the micropapillary type compared to that of typical ovarian serous borderline tumors: radiologic-pathologic correlation. J Ovarian Res.

[51] Deligdisch L, Pénault-Llorca F, Schlosshauer P (2007). Stage I ovarian carcinoma: different clinical pathologic patterns. Fertil Steril.

[52] Robinson KA, Menias CO, Chen L (2020). Understanding malignant transformation of endometriosis: imaging features with pathologic correlation. Abdom Radiol (NY).

[53] McCluggage WG (2014). Ovarian borderline tumours: a review with comparison of serous and mucinous types. Diagn Histopathol.

[54] Rutgers JKL (2016). Mullerian Mucinous/Mixed Epithelial (Seromucinous) Ovarian Tumors. AJSP Rev Rep.

[55] Kurata Y, Kido A, Moribata Y (2017). Diagnostic performance of MR imaging findings and quantitative values in the differentiation of seromucinous borderline tumour from endometriosis-related malignant ovarian tumour. Eur Radiol.

[56] Revzin MV, Moshiri M, Katz DS (2020). Imaging Evaluation of Fallopian Tubes and Related Disease: A Primer for Radiologists. Radiographics.

[57] Kier R (1992). Nonovarian gynecologic cysts: MR imaging findings. AJR Am J Roentgenol.

[58] Chaudhari VV, Patel MK, Douek M, Raman SS (2010). MR Imaging and US of Female Urethral and Periurethral Disease. Radiographics.

[59] Aldrich ER, Pauls RN (2021). Benign Cysts of the Vulva and Vagina: A Comprehensive Review for the Gynecologic Surgeon. Obstet Gynecol Surv.

[60] Zulfiqar M, Shetty A, Yano M (2021). Imaging of the Vagina: Spectrum of Disease with Emphasis on MRI Appearance. Radiographics.

[61] Hosseinzadeh K, Heller MT, Houshmand G (2012). Imaging of the Female Perineum in Adults. Radiographics.

[62] Hosseinzadeh K, Furlan A, Torabi M (2008). Pre- and postoperative evaluation of urethral diverticulum. AJR Am J Roentgenol.

[63] Noiret B, Renaud F, Piessen G, Eveno C (2019). Multicystic peritoneal mesothelioma: a systematic review of the literature. Pleura Peritoneum.

[64] Kepenekian V, Péron J, Goéré D (2021). Multicystic peritoneal mesothelioma treated with cytoreductive surgery followed or not by hyperthermic intraperitoneal chemotherapy: results from a large multicentric cohort. Int J Hyperthermia.

[65] Low RN, Barone RM, Rousset P (2021). Peritoneal MRI in patients undergoing cytoreductive surgery and HIPEC: History, clinical applications, and implementation. Eur J Surg Oncol.

[66] Brown IS, Sokolova A, Rosty C, Graham RP (2023). Cystic lesions of the retrorectal space. Histopathology.

[67] Hjermstad BM, Helwig EB (1988). Tailgut cysts. Report of 53 cases. Am J Clin Pathol.

[68] Lev-Chelouche D, Gutman M, Goldman G (2003). Presacral tumors: a practical classification and treatment of a unique and heterogeneous group of diseases. Surgery.

[69] Dahan H, Arrivé L, Wendum D (2001). Retrorectal developmental cysts in adults: clinical and radiologic-histopathologic review, differential diagnosis, and treatment. Radiographics.

[70] Moyle PL, Kataoka MY, Nakai A (2010). Nonovarian Cystic Lesions of the Pelvis <sup/>. Radiographics.

[71] Aflalo-Hazan V, Rousset P, Mourra N (2008). Tailgut cysts: MRI findings. Eur Radiol.

[72] Burke JR, Shetty K, Thomas O (2022). The management of retrorectal tumours: tertiary centre retrospective study. BJS Open.

[73] Parada Villavicencio C, Adam SZ, Nikolaidis P (2016). Imaging of the Urachus: Anomalies, Complications, and Mimics. Radiographics.

[74] Cappele O, Sibert L, Descargues J (2001). A study of the anatomic features of the duct of the urachus. Surg Radiol Anat.

[75] Rafat Zand K, Reinhold C, Haider MA (2007). Artifacts and pitfalls in MR imaging of the pelvis. J Magn Reson Imaging.

[76] Chabrol A, Rousset P, Charlot M (2014). Lesions of the ovary with T1-hypersignal. Clin Radiol.

[77] Ginat DT, Meyers SP (2012). Intracranial Lesions with High Signal Intensity on T1-weighted MR Images: Differential Diagnosis. Radiographics.

[78] Delfaut EM, Beltran J, Johnson G (1999). Fat Suppression in MR Imaging: Techniques and Pitfalls. Radiographics.

[79] Ram R, Sarver D, Pandey T (2016). Magnetic resonance enterography: A stepwise interpretation approach and role of imaging in management of adult Crohn’s disease. Indian J Radiol Imaging.

[80] Griffin N, Grant LA, Sala E (2008). Magnetic resonance imaging of vaginal and vulval pathology. Eur Radiol.

