# Supplementary material for: Endometriosis MR mimickers: T1-hyperintense lesions
Source: Insights Imaging. 2024 Jan 24;15:19. doi: 10.1186/s13244-023-01587-3 (PMC10808095; doi:10.1186/s13244-023-01587-3)
Supplement: Supplementary file 1 — Additional file 1. [file 13244_2023_1587_MOESM1_ESM.docx]

# Endometriosis MR Mimickers: T1-Hyperintense Lesions

# ELECTRONIC SUPPLEMENTARY MATERIAL
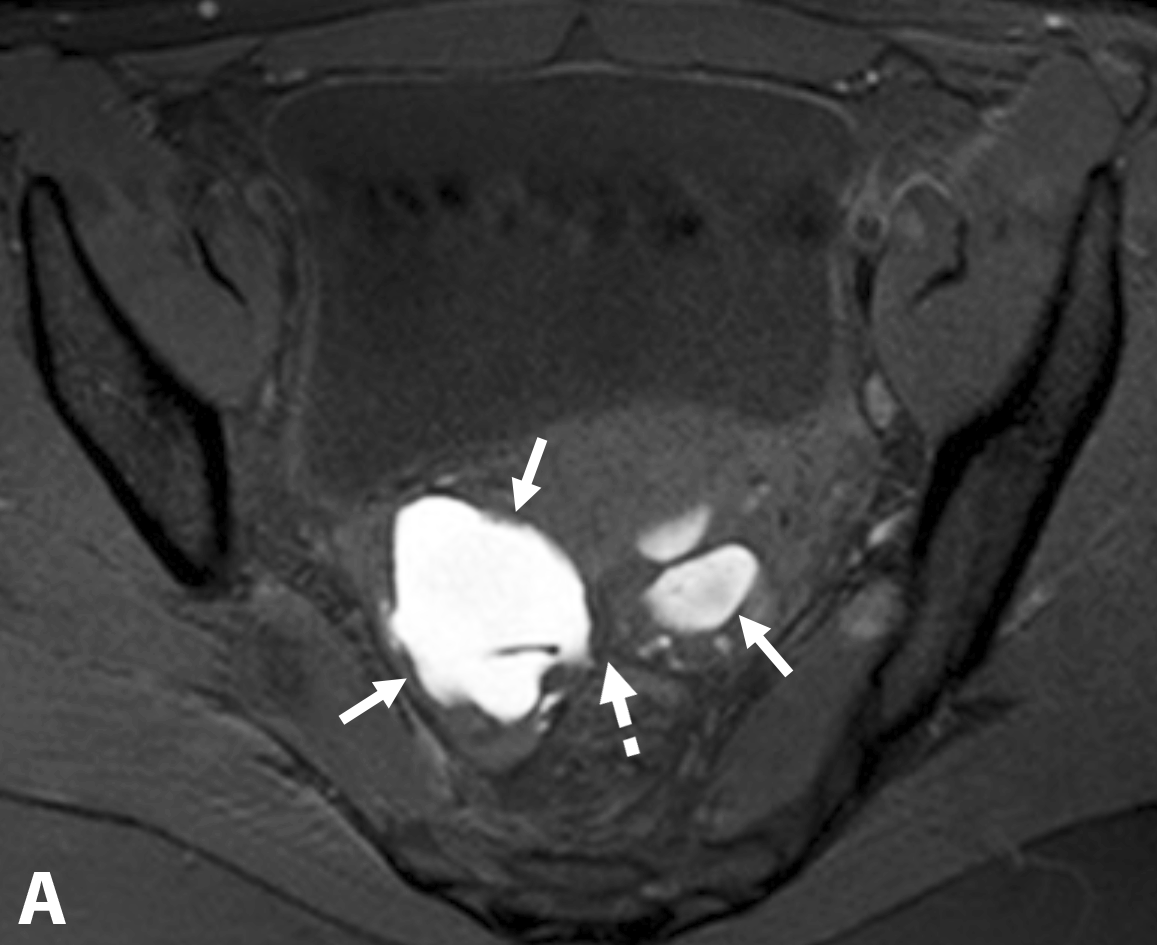

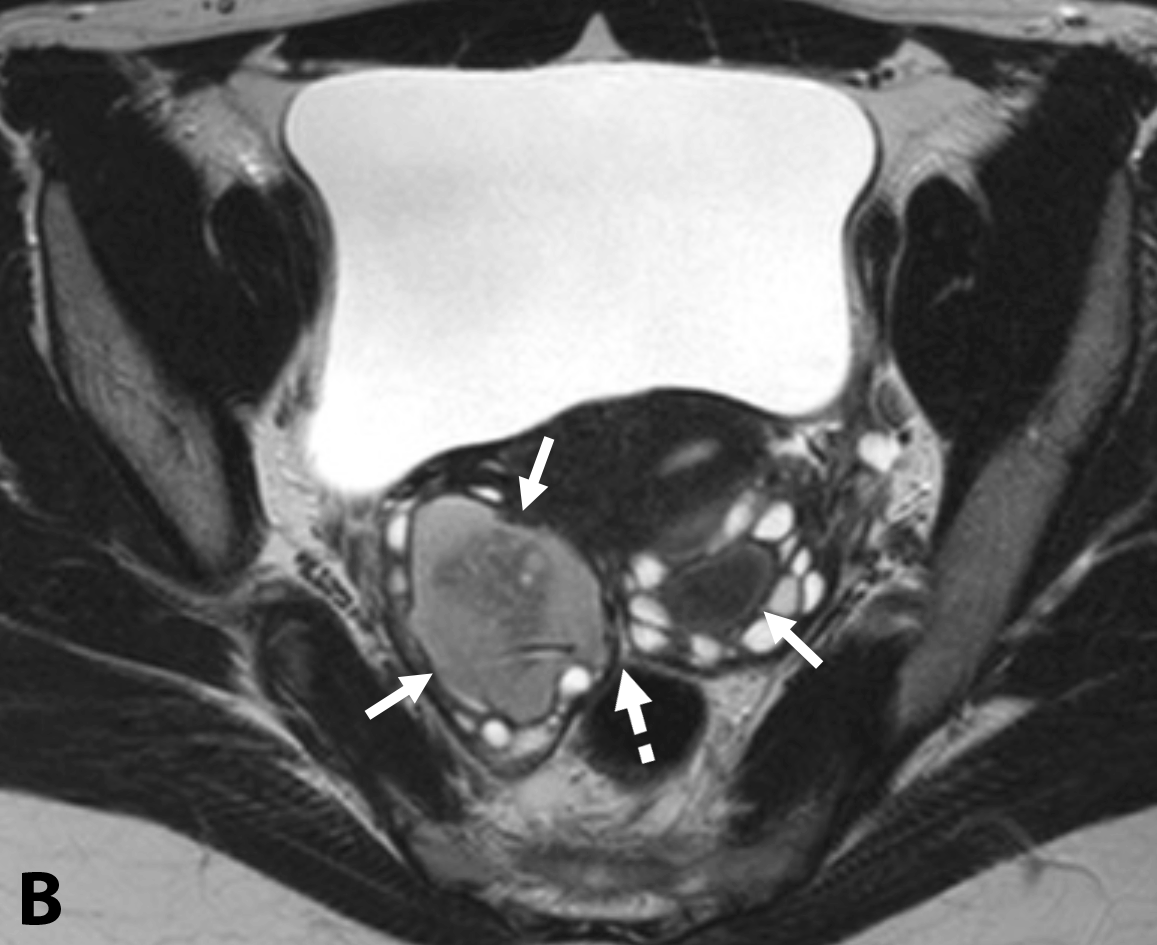

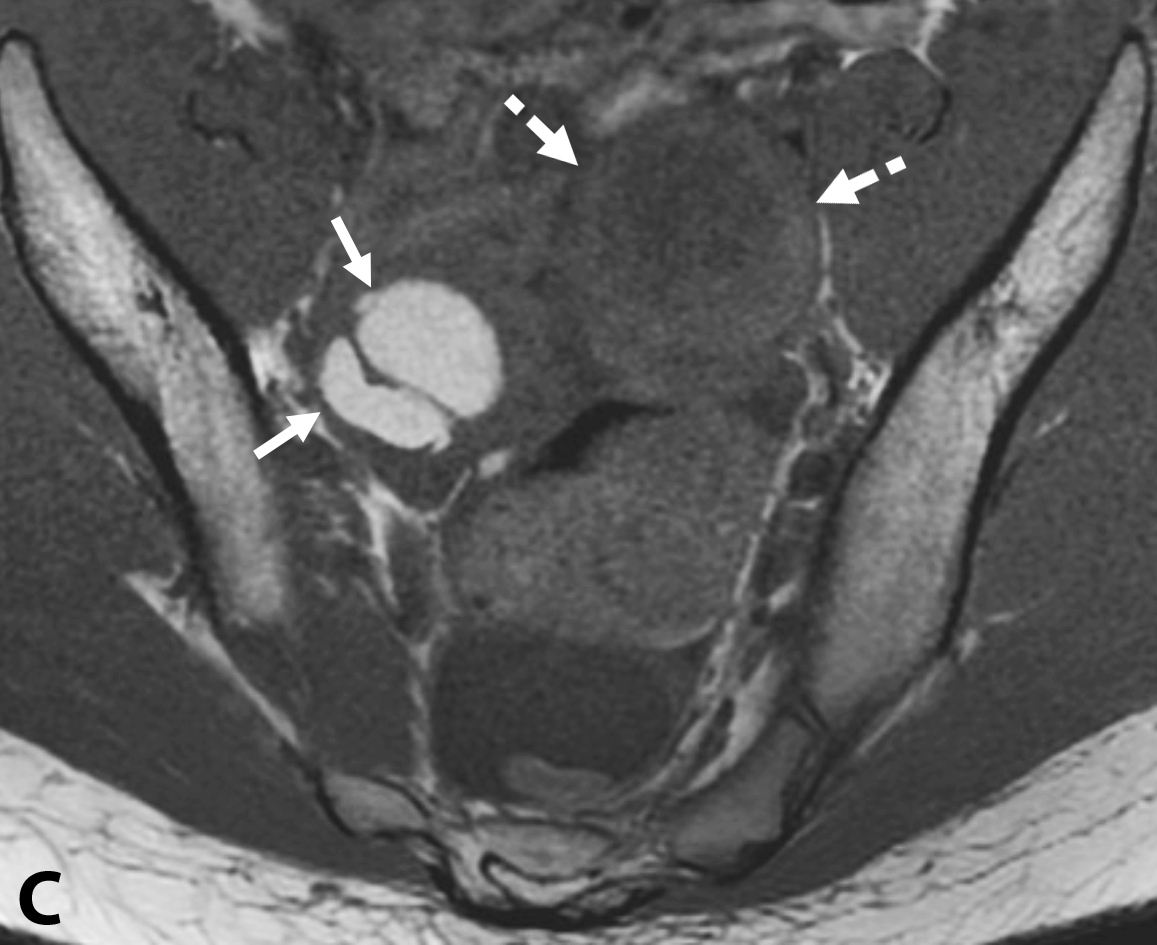

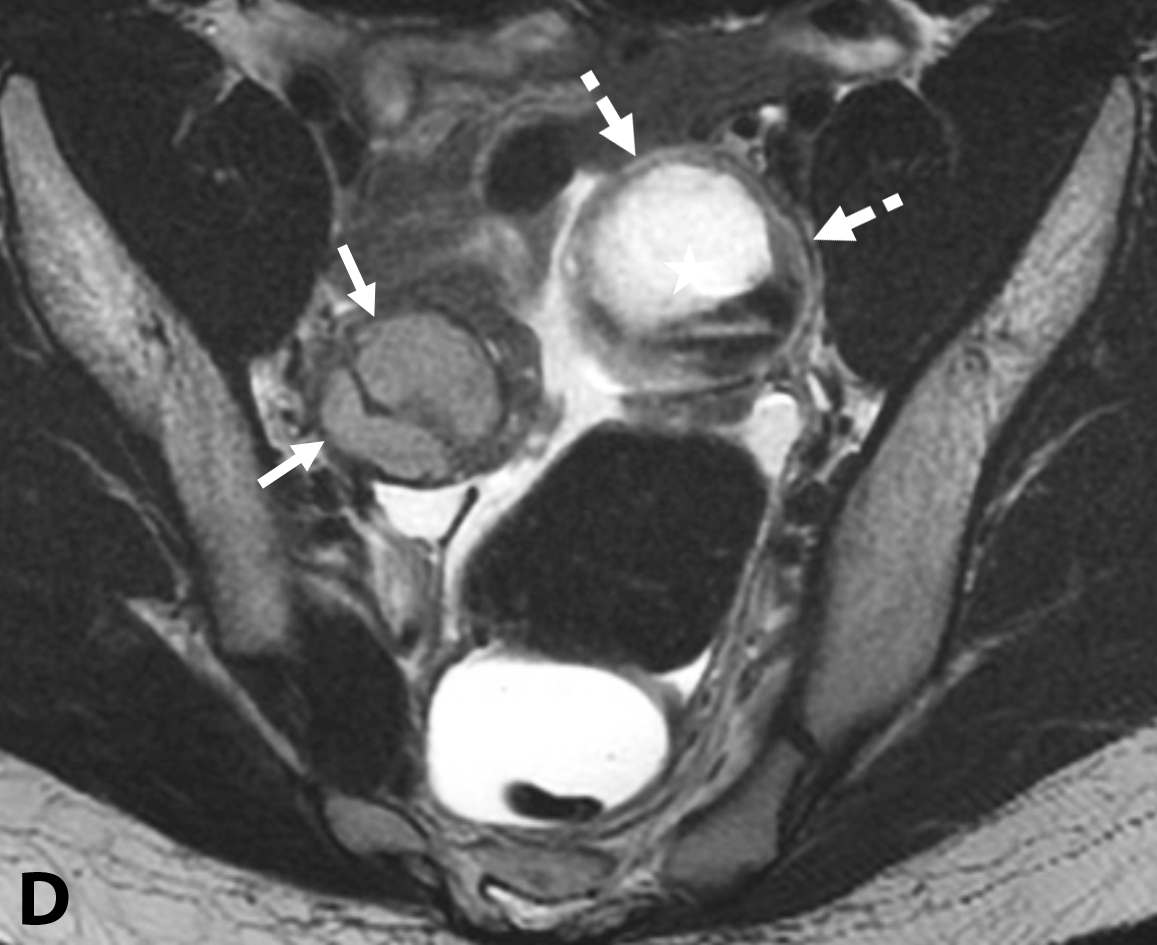

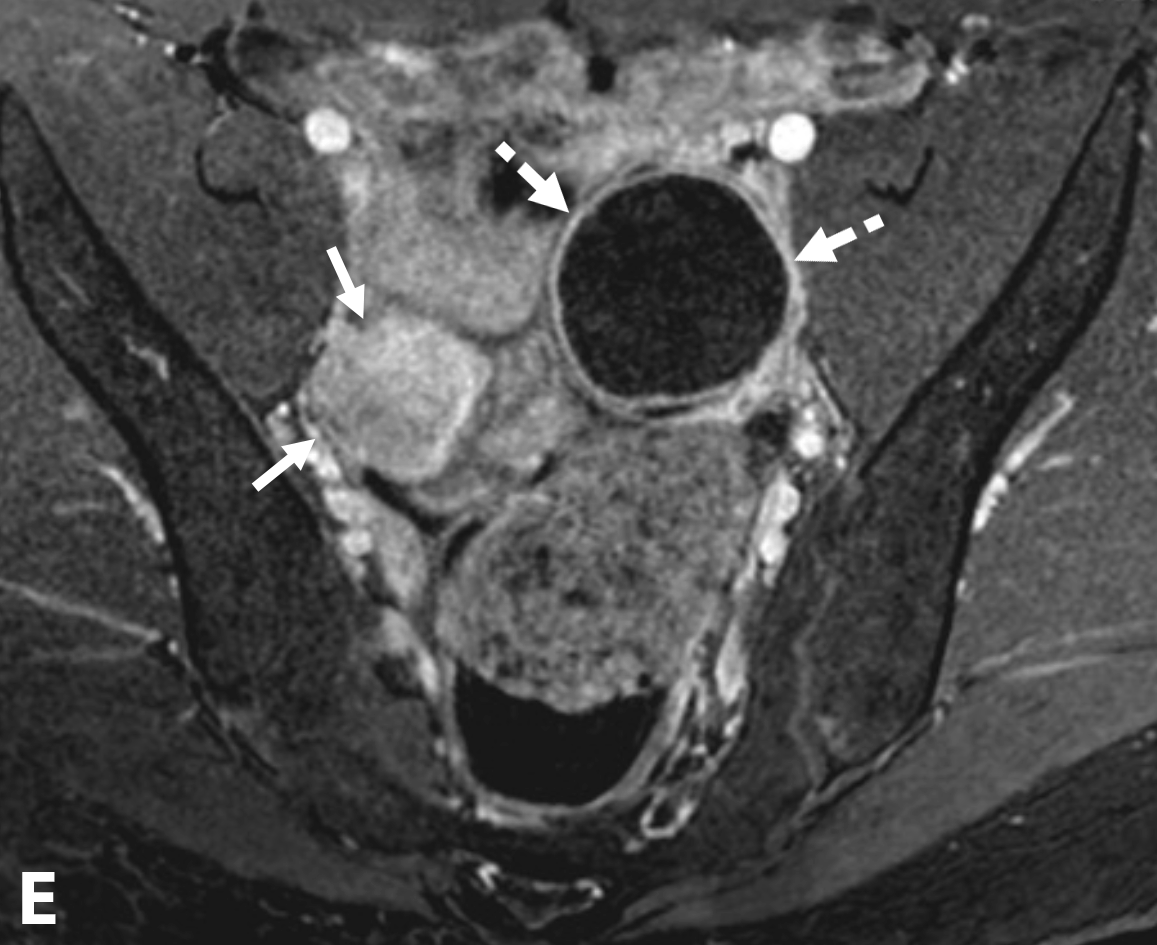


**Figure 1** - MRI key features of endometriosis in reproductive-age women with documented history of peep Pelvic endometriosis and endometriomas.

(a) Axial T1-W fat-suppressed and (b) axial T2-W MR images show bilateral endometriomas with T2-shading sign (arrows). Additionally, both ovaries are displaced medially and posteriorly in the retrocervical region (dashed arrows), defining a "kissing ovaries" sign.

(c) Axial T1-W, (d) axial T2-W, and (e) axial T1-W fat-suppressed contrast-enhanced subtracted MR images show a T1-hyperintense endometrioma (arrows) with T2-shading sign in the right ovary and displaying no enhancement around or within the endometrioma. Of note, the presence of a left-sided hemorrhagic ovarian cyst (dashed arrows) showing T2 heterogeneity, T1 iso-intensity, and a rim sign consisting of a peripheral enhancement.


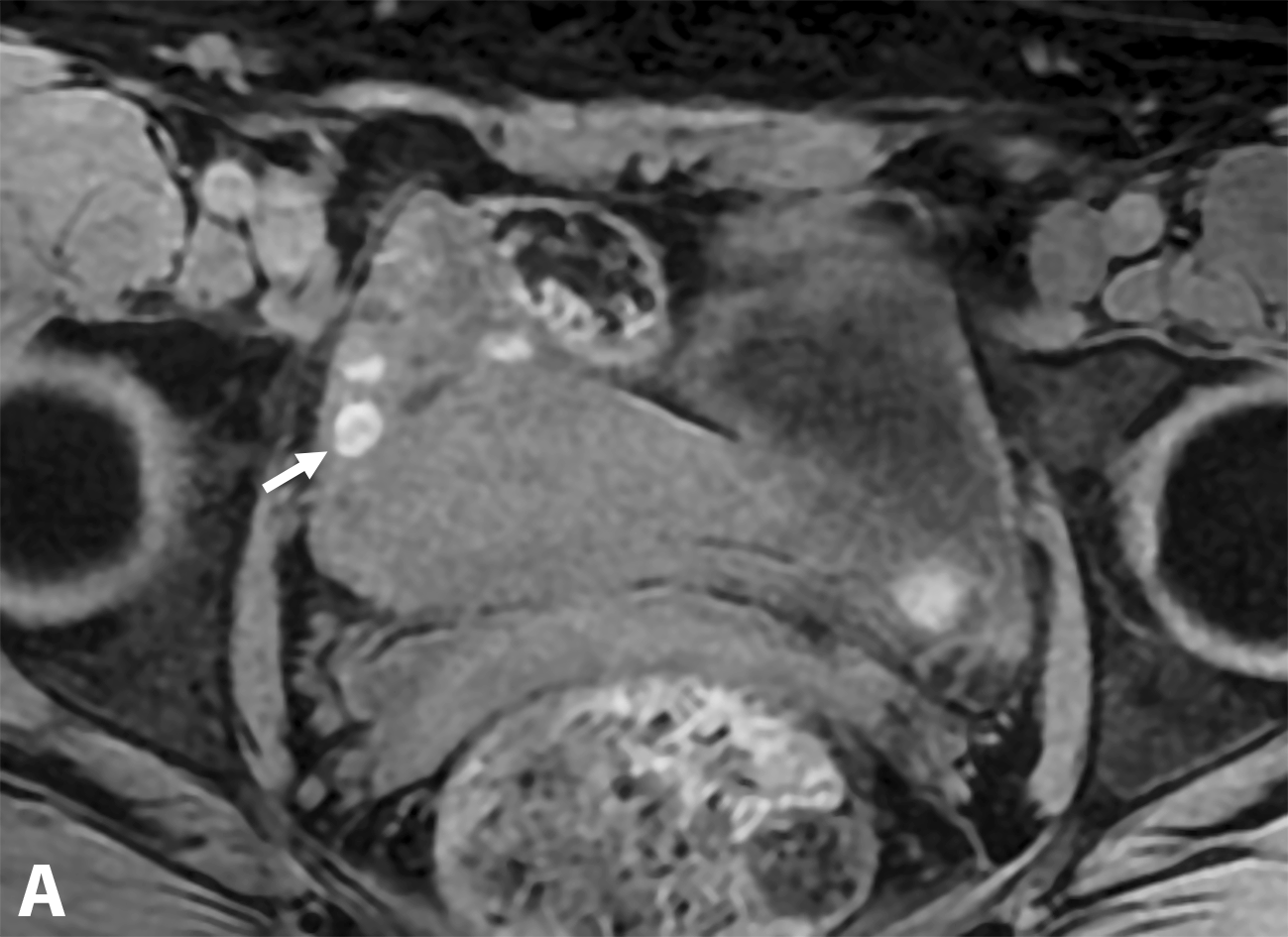

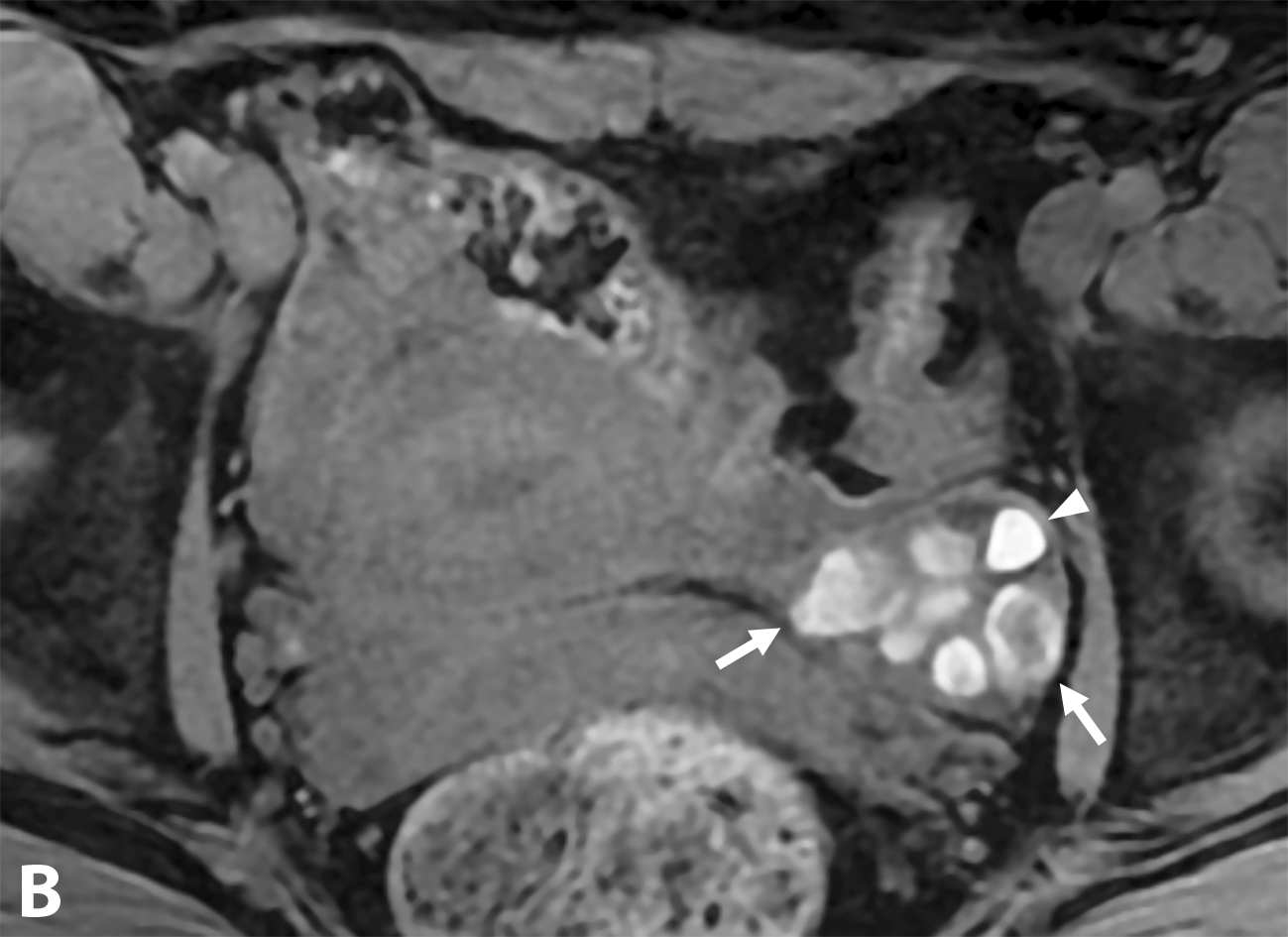

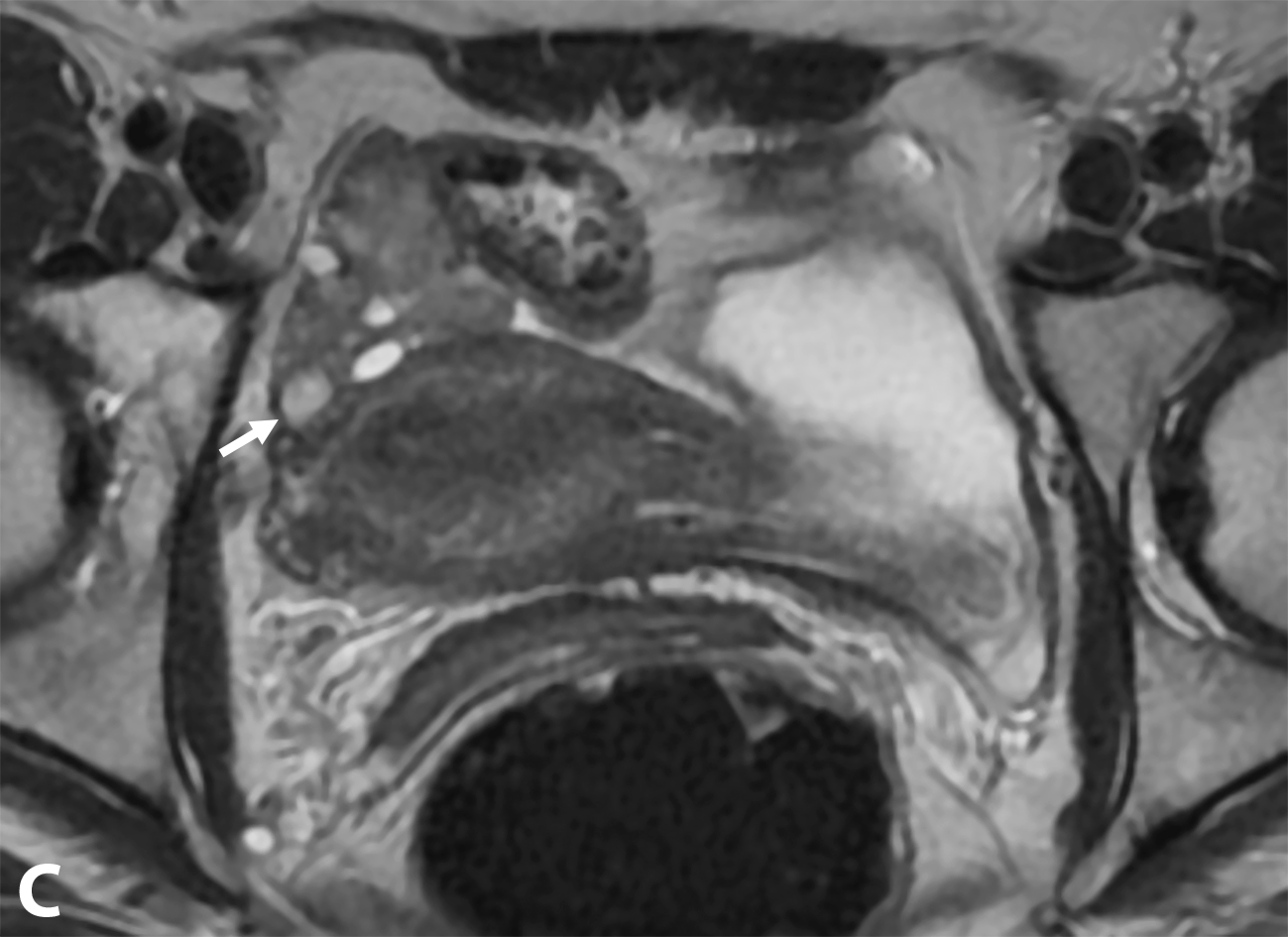

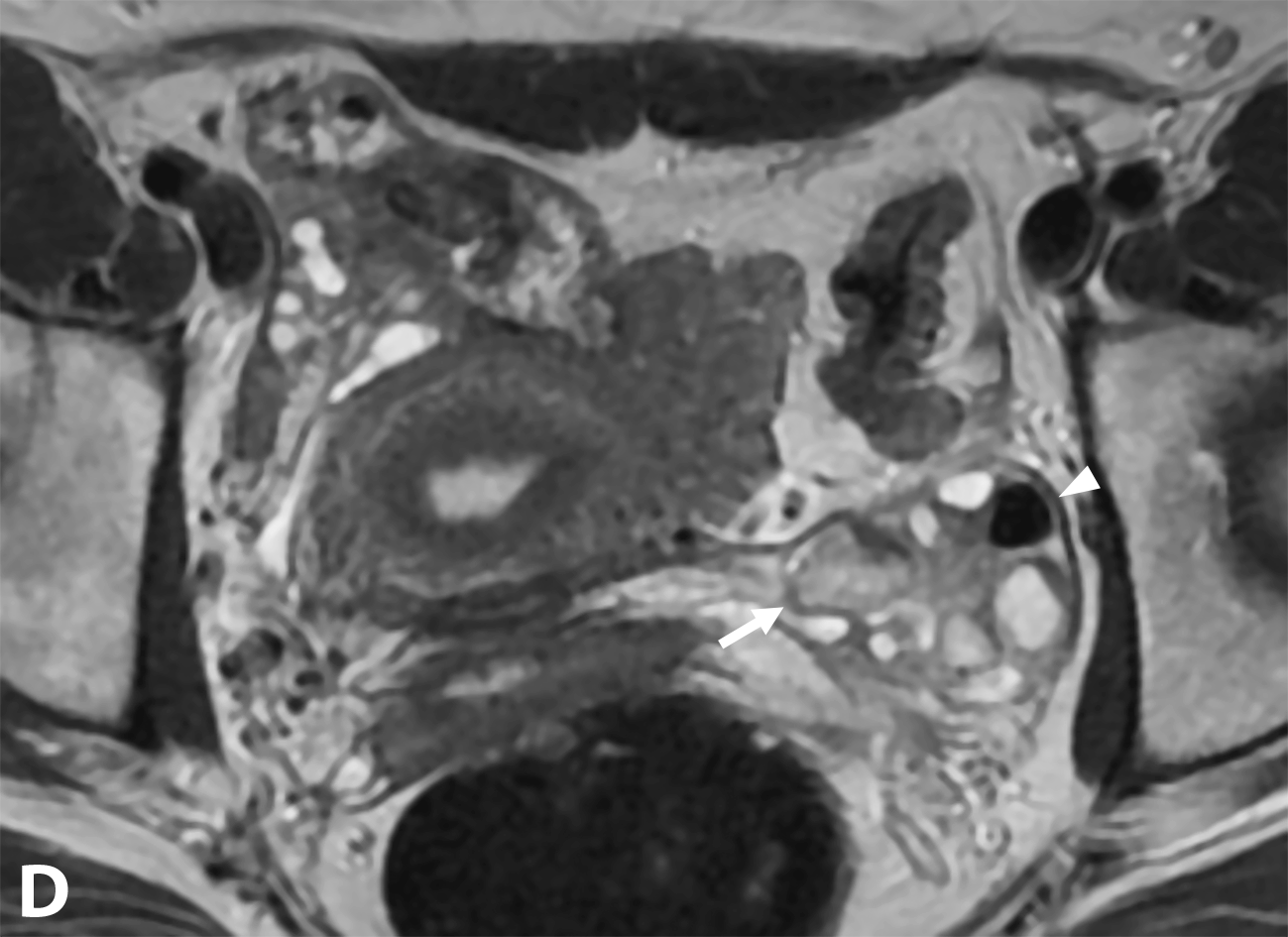


**Figure 2** - Hemorrhagic ovarian cysts – 29 days after follicular puncture - in a 32-year-old woman with documented medical history of deep pelvic endometriosis and endometriomas.

(a, b) Axial T1-W fat-suppressed MR images show T1-hyperintense hemorrhagic cysts in both ovaries, with various signal intensities (arrows).

(c, d) Axial T2-W MR images show multiple fluid-filled cystic ovarian lesions (arrows), with low (T2 shading) to high T2-signal intensity.

One cyst in the left ovary shows higher T1-hyperintensity and T2 shading effect (arrowheads), which may be more consistent with a micro-endometrioma.


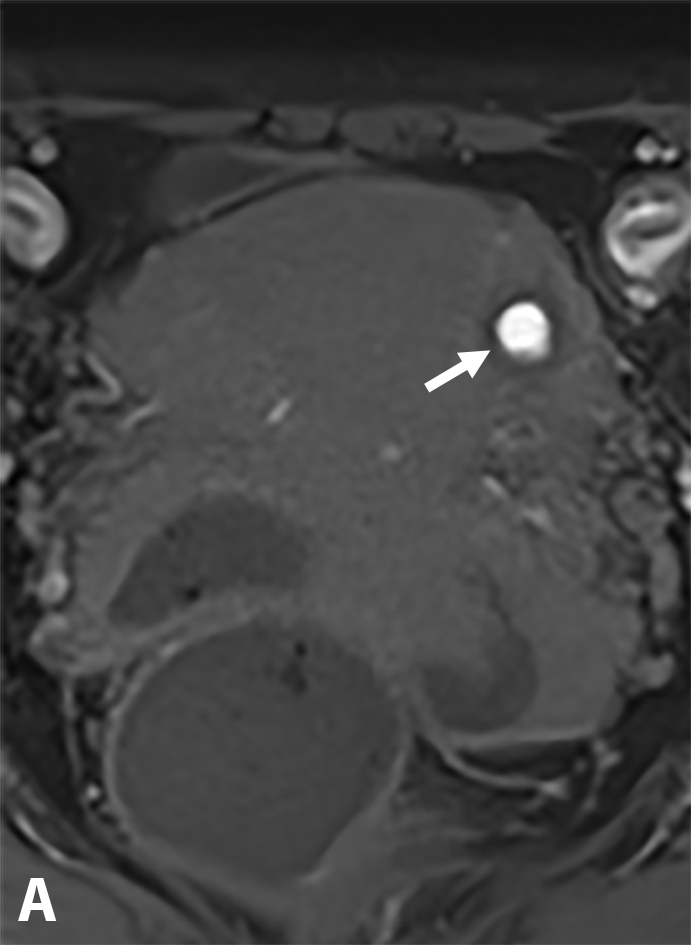

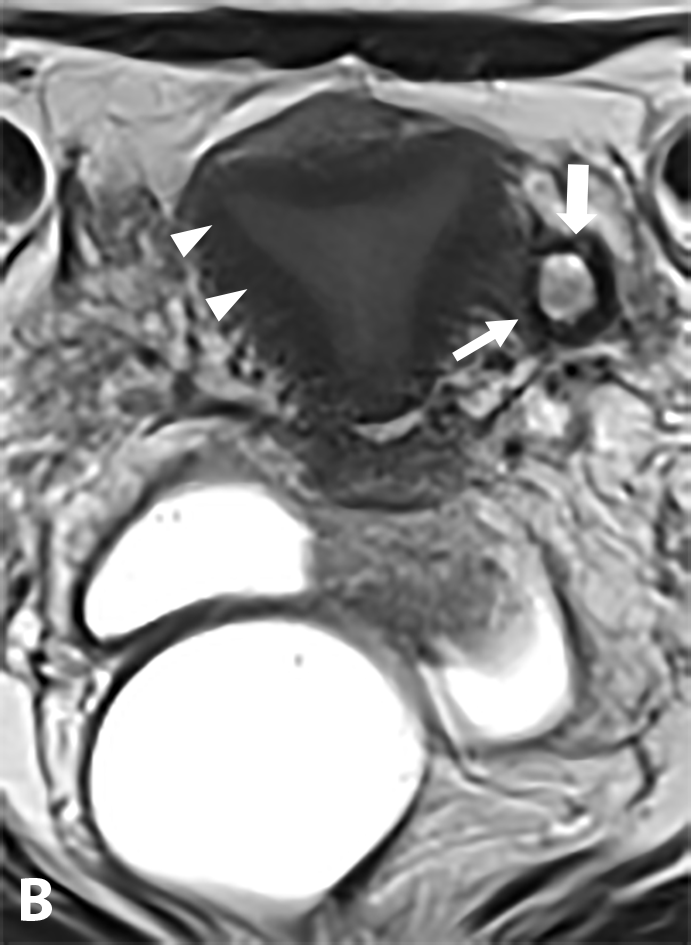

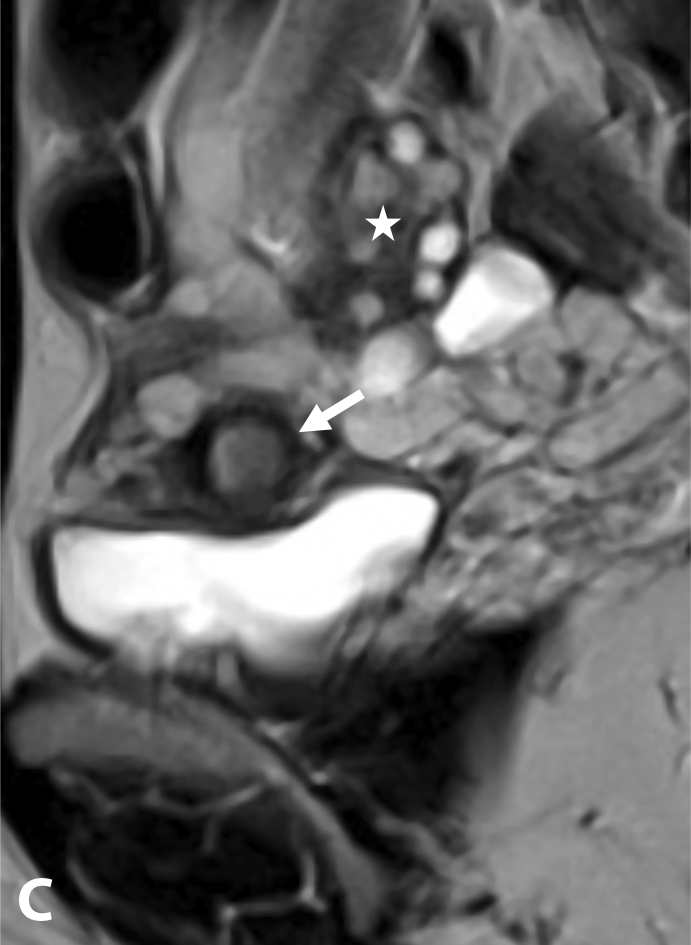


**Figure 3** - Accessory cavitated uterine mass (ACUM) in the left broad ligament in a 35-year-old woman with severe dysmenorrhea and left sided chronic pelvic pain, resistant to treatment.

(a) Axial T1-W fat-suppressed MR image shows a T1-hyperintense left sided central cavity (thin arrow), suggestive of hemorrhagic content.

(b) Axial and (c) sagittal T2-W MR images show a left-sided mass in the broad ligament with intermediate T2-signal intensity within its central cavity (thick arrow) surrounded by a T2-hypointense peripheral ring (thin arrow), identical to the uterine junctional zone (arrowheads). Note the presence of adjacent but distinct left ovary (star).

**
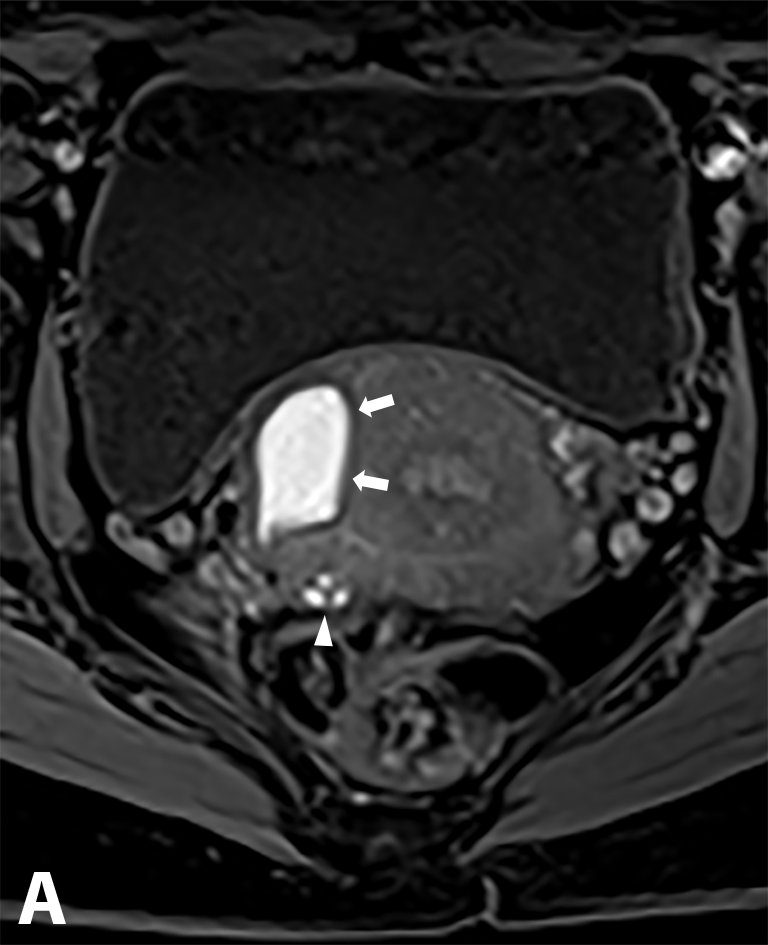

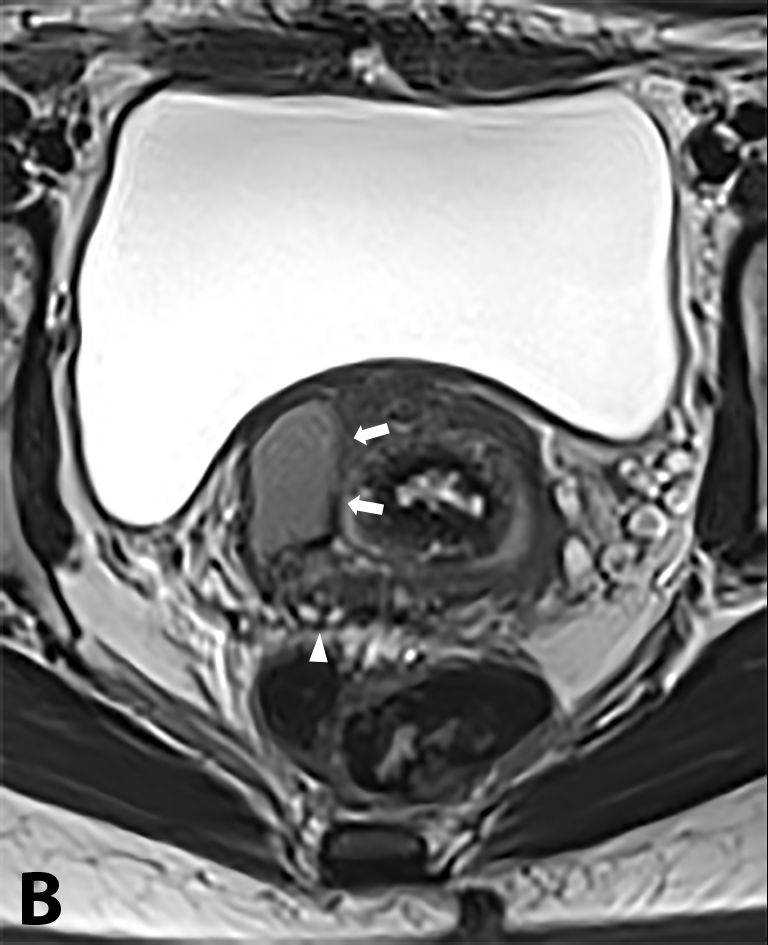
**

**Figure 4** - Gartner’s duct cyst in a 29-year-old woman with deep pelvic endometriosis.

(a) Axial T1-W fat-suppressed MR image shows a T1-hyperintense right vaginal cyst (arrows), and multiple hemorrhagic foci within the posterior vaginal fornix (arrowhead).

(b) Axial T2-W MR images shows a T2-intermediate signal intensity (T2 shading) right vaginal cyst (arrows) with thin walls, and deep infiltrating endometriosis lesion of the posterior vaginal fornix (arrowhead) extending from the right uterosacral ligament.

At surgery, pelvic endometriosis and Gartner’s duct cyst were removed.


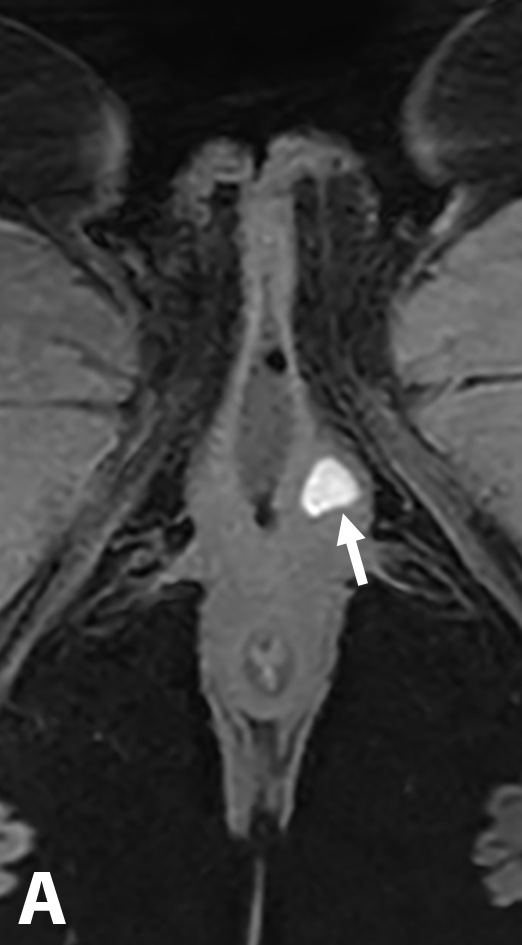

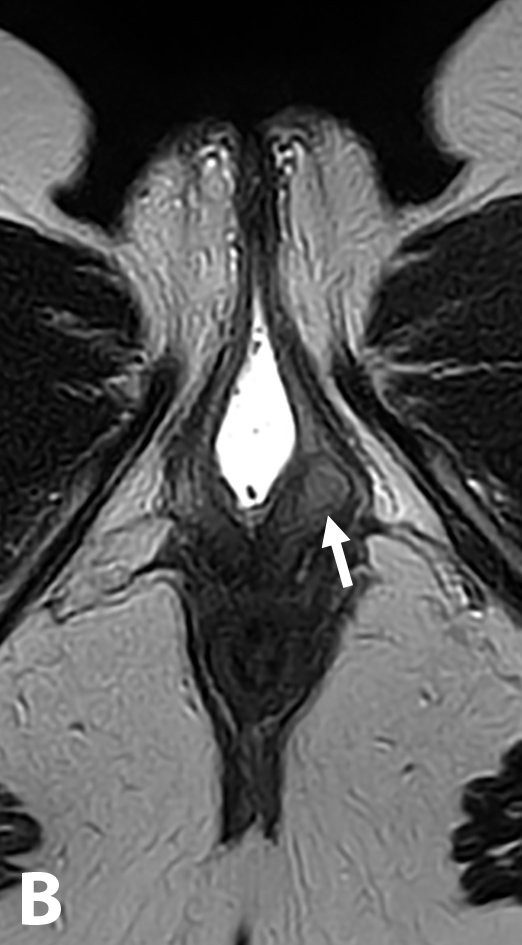

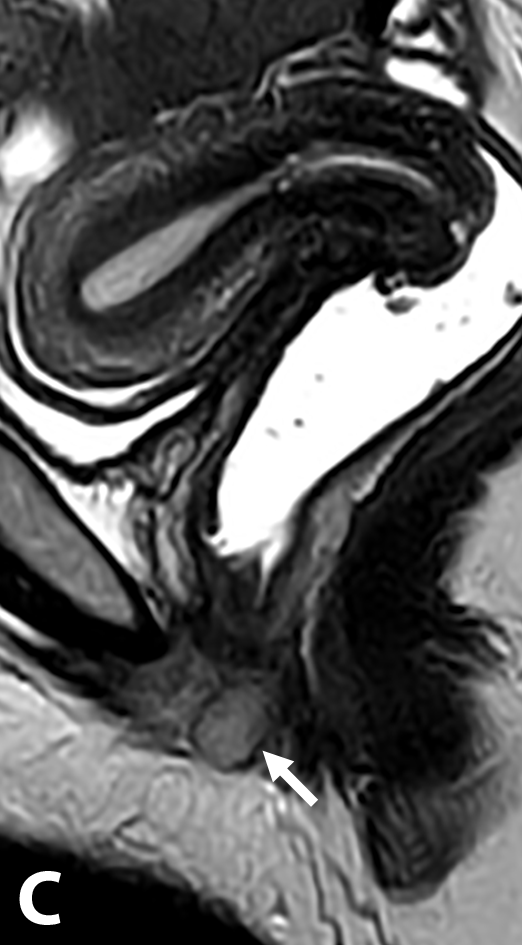


**Figure 5** - Bartholin’s gland cyst in a 37-year-old woman.

(a) Axial T1-W fat-suppressed, (b) axial and (c) sagittal T2-W MR images show a T1-hyperintense and T2 intermediate signal intensity thin-walled cystic lesion (arrows) along the left aspect of the vulva.


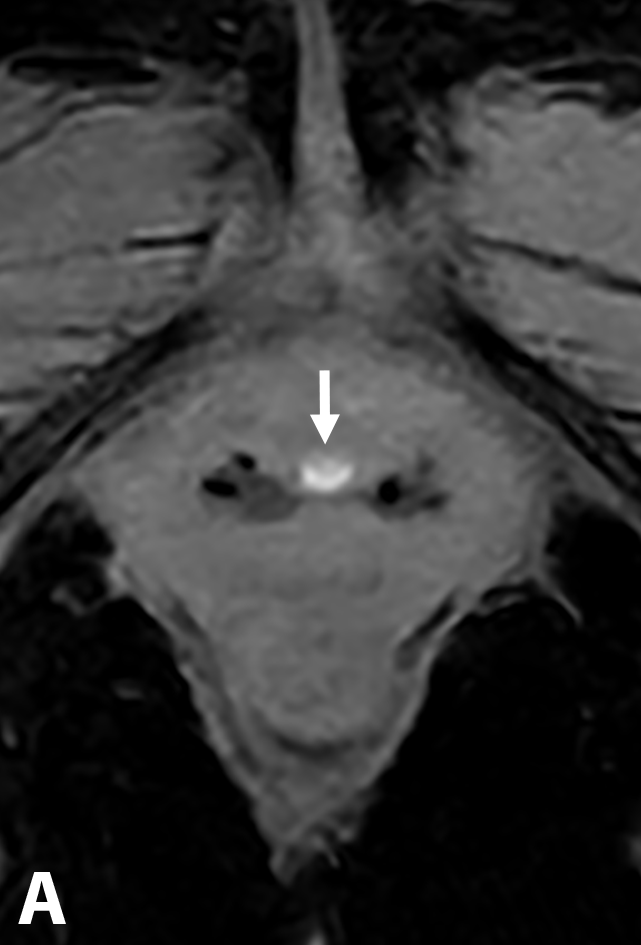

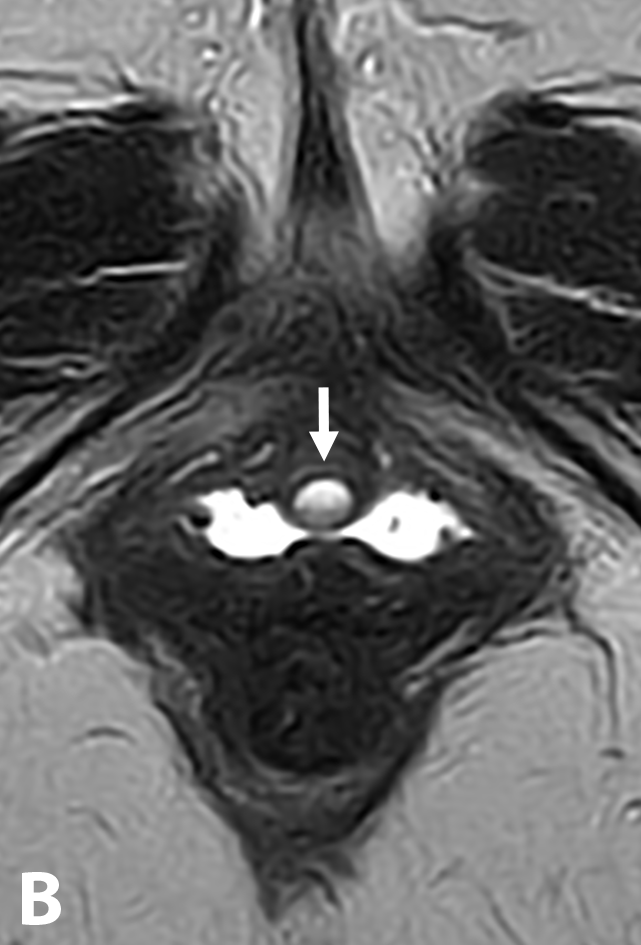

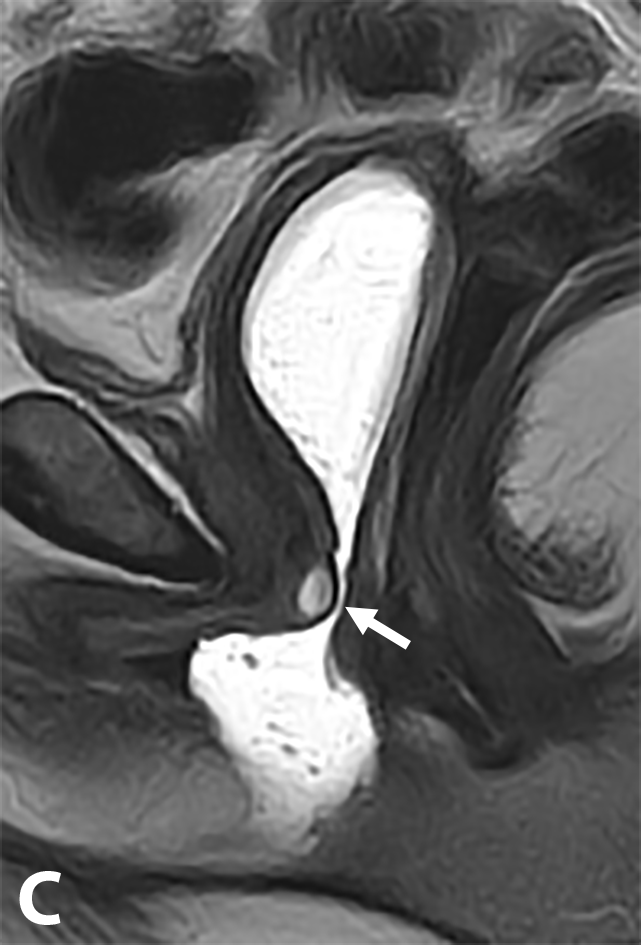


**Figure 6** - Skene’s gland cyst in a 46-year-old woman.

(a) Axial T1-W fat-suppressed MR image shows a T1-hyperintense round cyst (arrow), located behind the external urethral meatus.

(b) Axial and (c) sagittal T2-W images show a T2-hyperintense cyst (arrow) with thin walls, along the external urethral meatus, anterior to the vagina. Note the T2 shading effect in the declive part of the cyst.

**
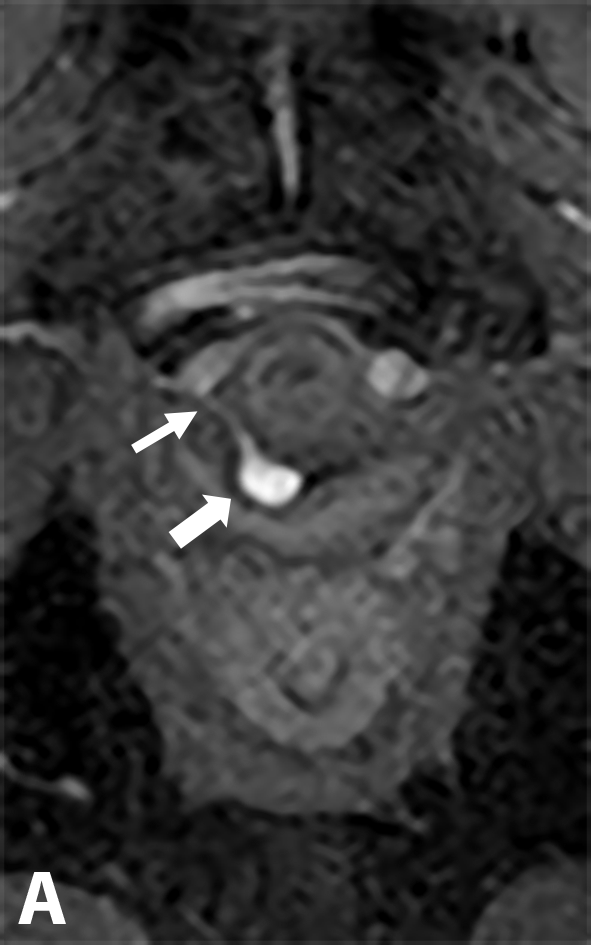

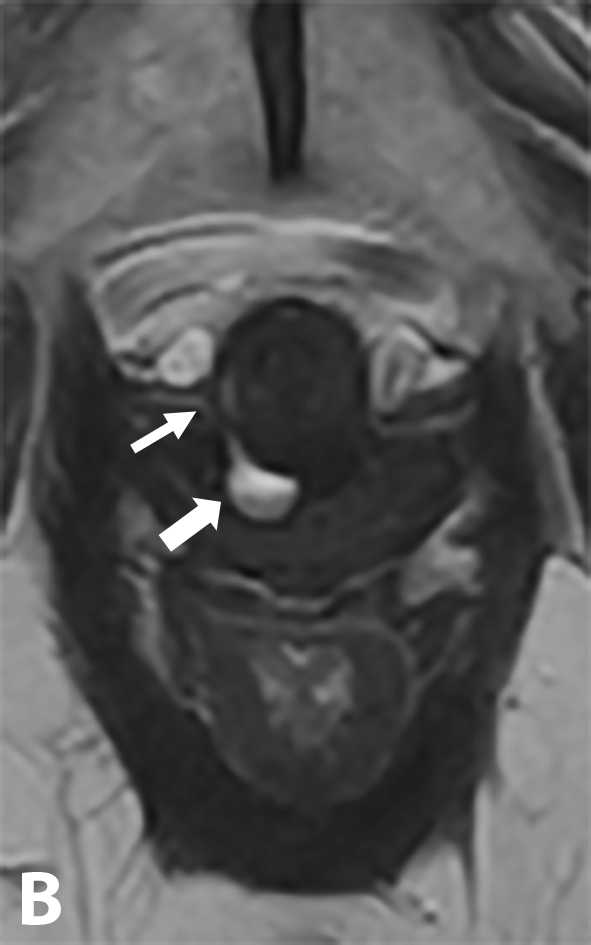

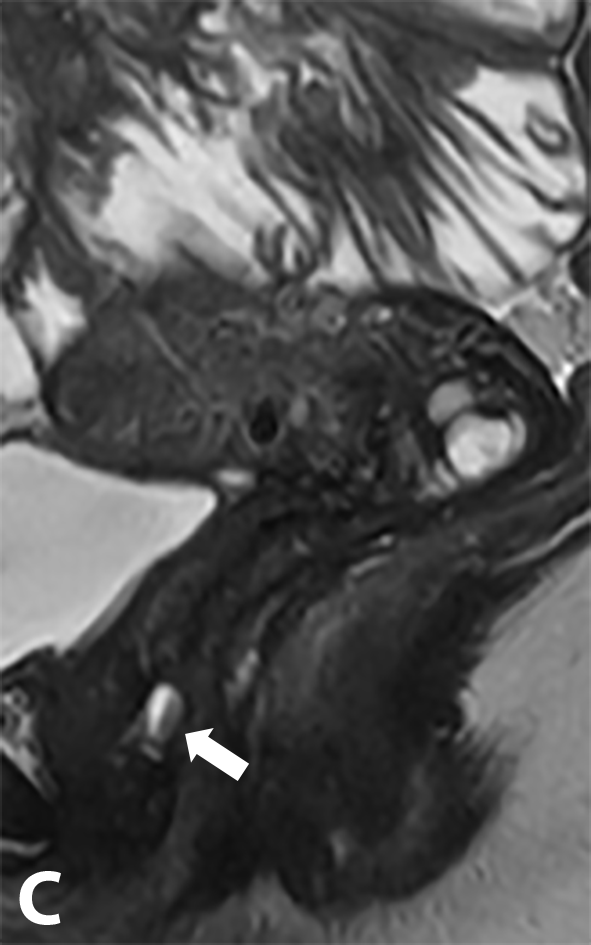
**

**Figure 7** - Urethral diverticulum in a 48-year-old woman with past medical history of uterine polyp resection.

(a) Axial T1-W fat-suppressed, (b) axial and (c) sagittal T2-W MR images show a T1-hyperintense and T2-hyperintense unilocular cyst (thick arrows) with fluid-fluid level and an opening tail (thin arrows) emerging from posterolateral mid/distal urethra.

**
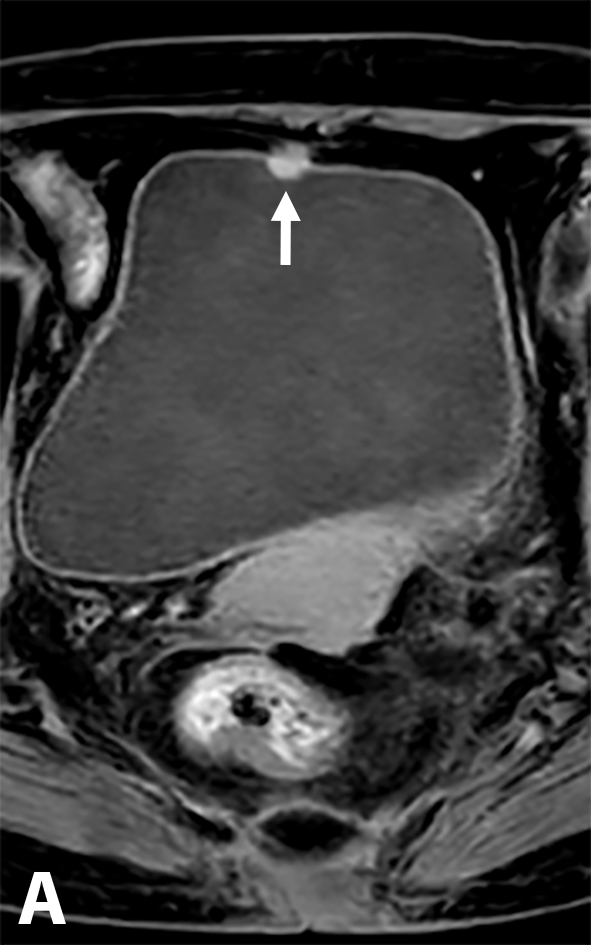

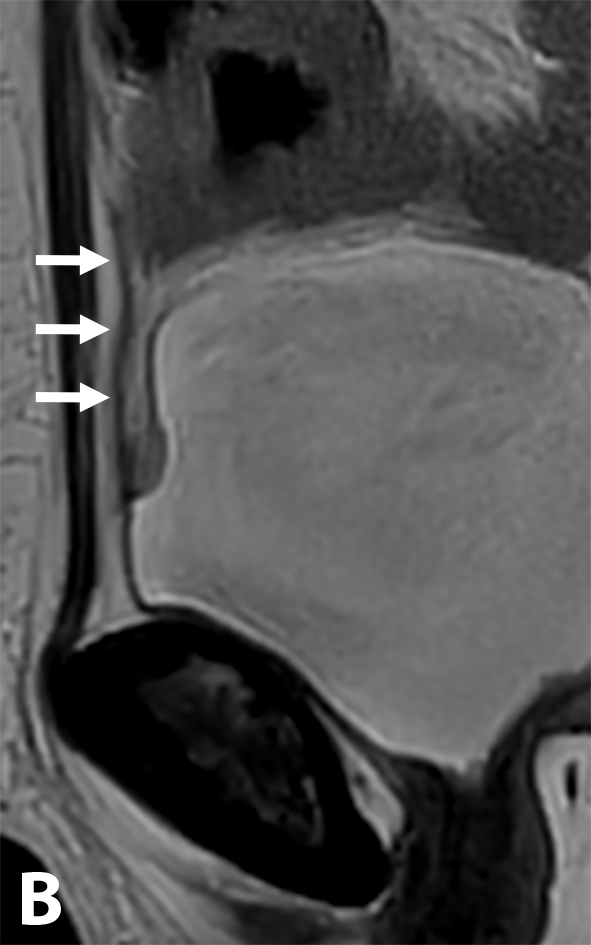

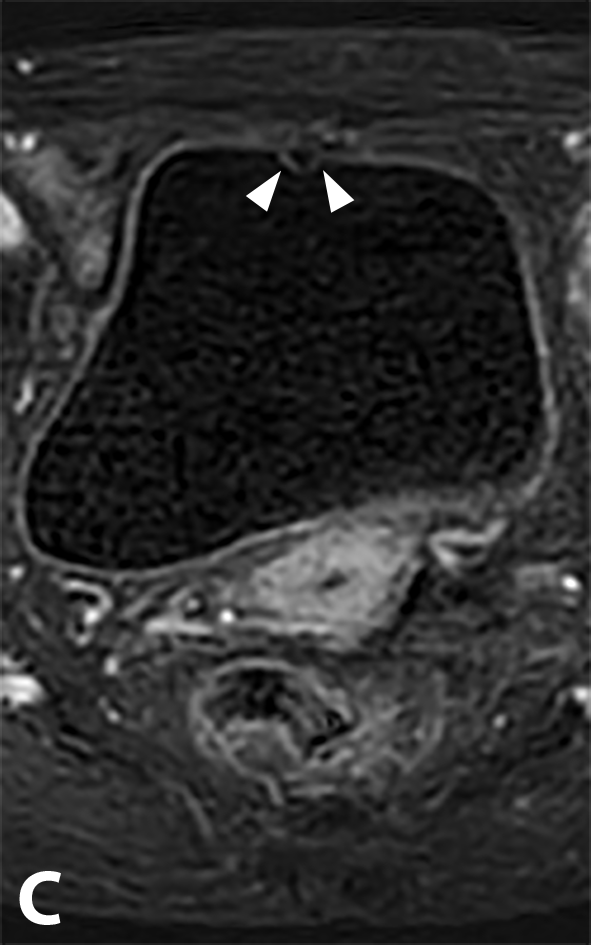
**

**Figure 8** - Urachus cystic remnant in a 36-year-old woman.

(a) Axial T1-W fat-suppressed MR image shows a small-sized and well-defined T1-hyperintense cyst (arrow) of the anterosuperior bladder wall (bladder dome).

(b) Sagittal T2-W image shows a continuity of the cyst with the ascendant subperitoneal anatomical course of the urachus (arrows).

(c) Axial T1-W fat-suppressed contrast-enhanced subtracted MR image shows a slight enhancement of the cyst wall (arrowheads).


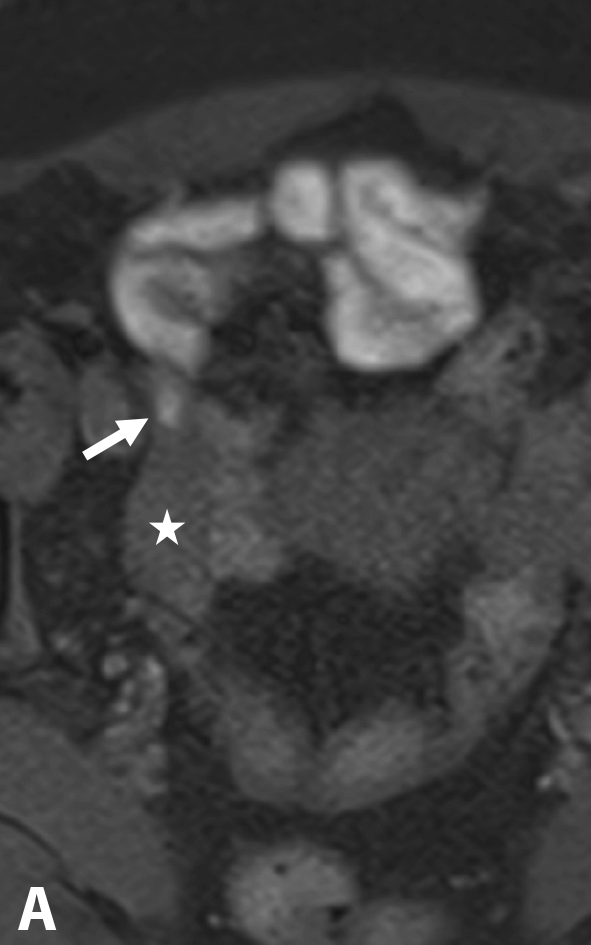

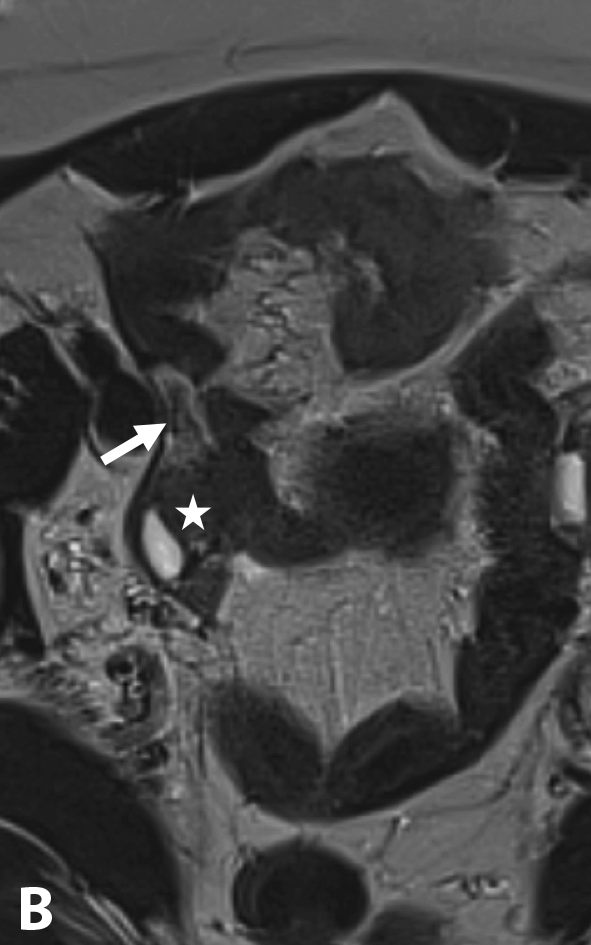

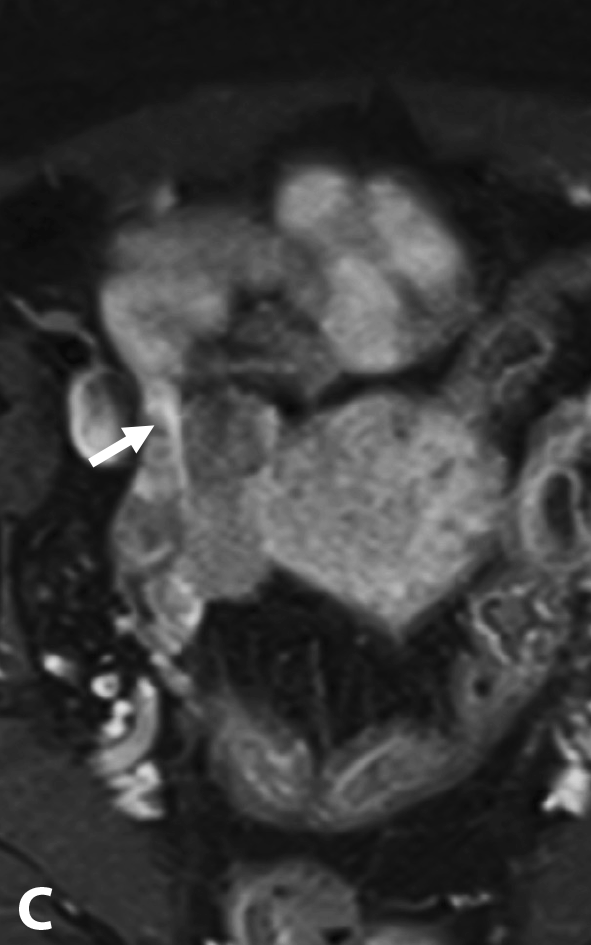


**Figure 9** - Flow-Related Enhancement artifact, also known as “the entry slice phenomenon”, in a 36-year-old woman.

(a) Axial T1-W fat-suppressed MR image shows a bright « pseudo-lesion » (arrow) forward and in contact with the right ovarian fossa (stars on a and b).

(b) Axial T2-W and (c) axial T1-W contrast-enhanced MR images show T2 partial flow-void (arrow on b) and linear gadolinium-enhancement (arrow on c) corresponding to the right ovarian vascular pedicle.


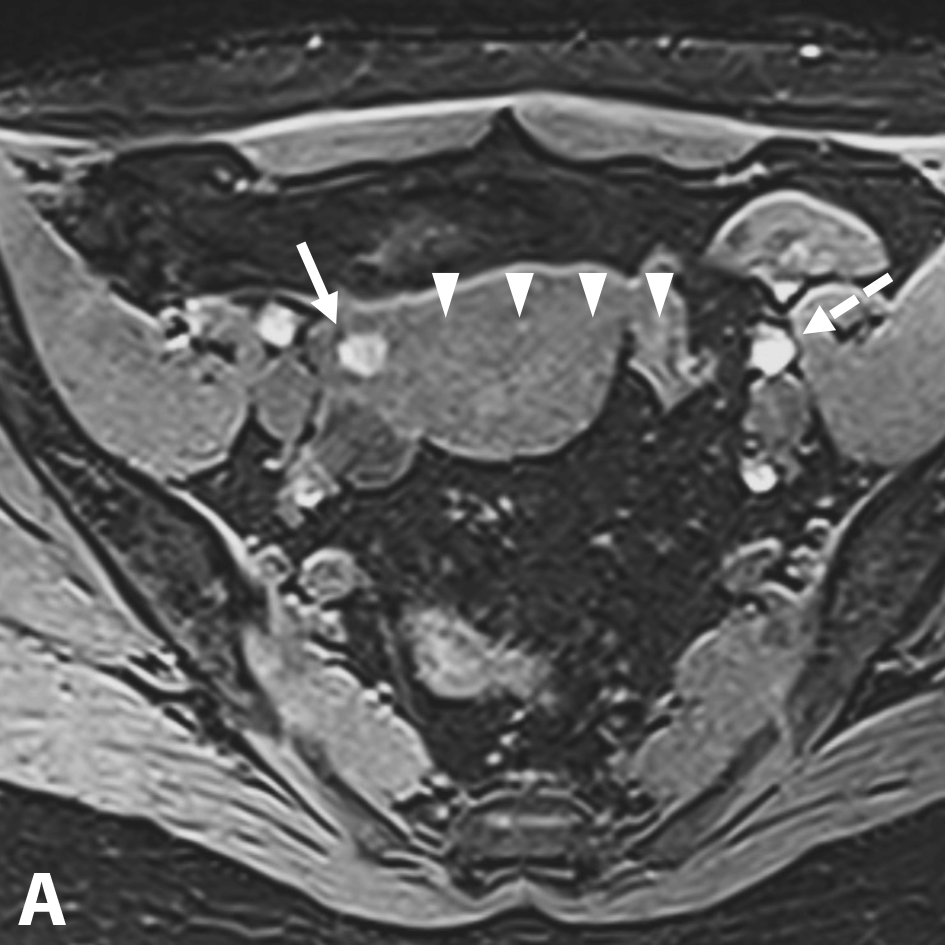

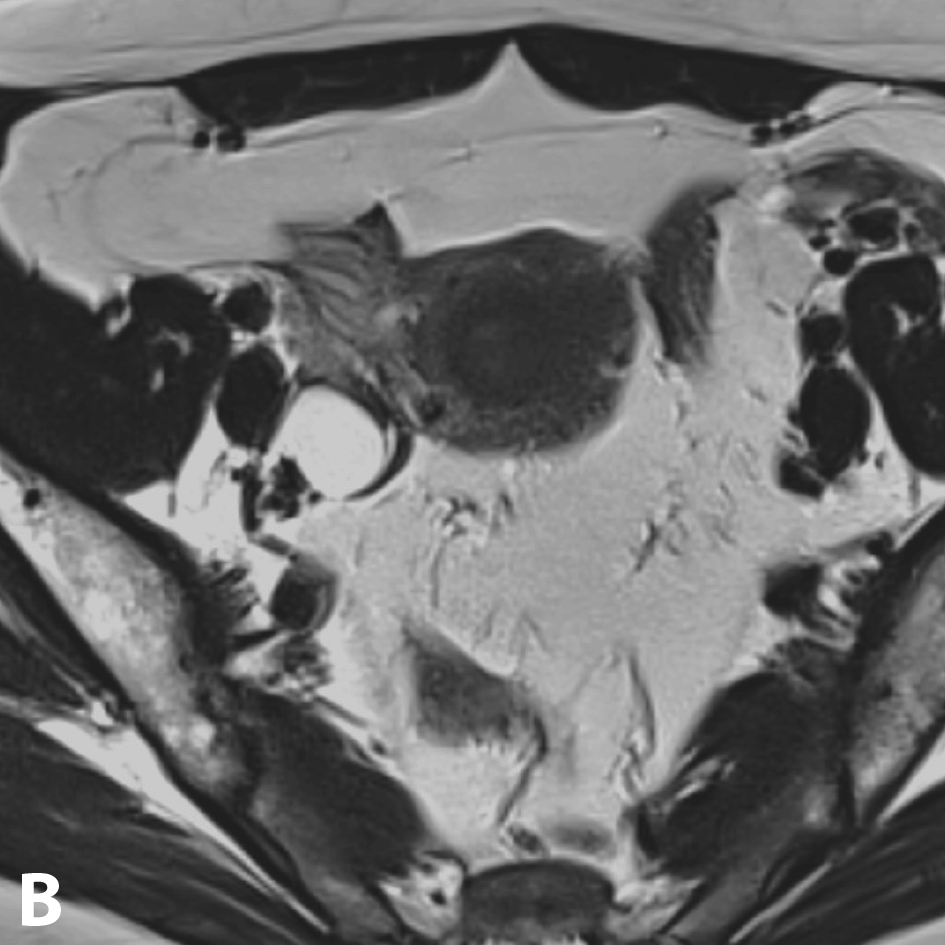


**Figure 10** - Vascular ghost artifact in a 23-year-old patient.

(a) Axial T1-W fat-suppressed MR image shows a T1-hyperintense round signal (thin arrow) on the right side of the uterus, on the same horizontal line projection (arrowheads) of left-sided external iliac arteries (dashed arrow).

(b) Axial T2-W MR image shows the absence of a corresponding lesion, without any abnormality of the myometrium and the right broad ligament.


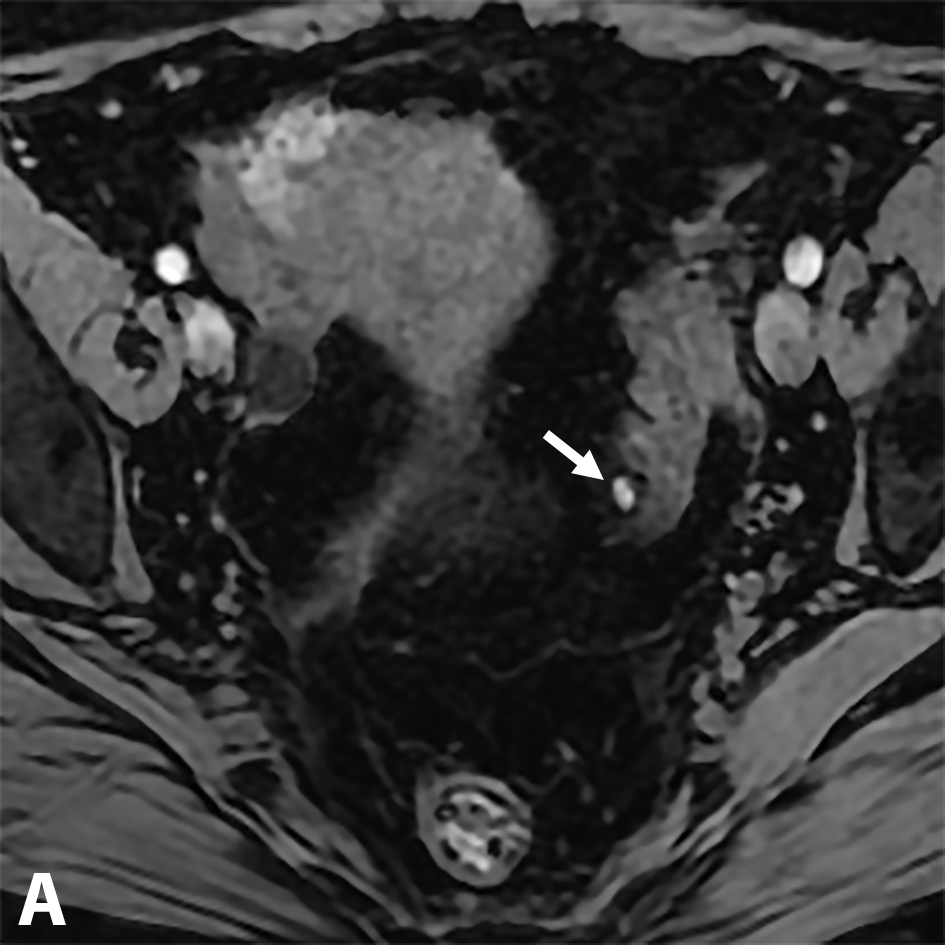

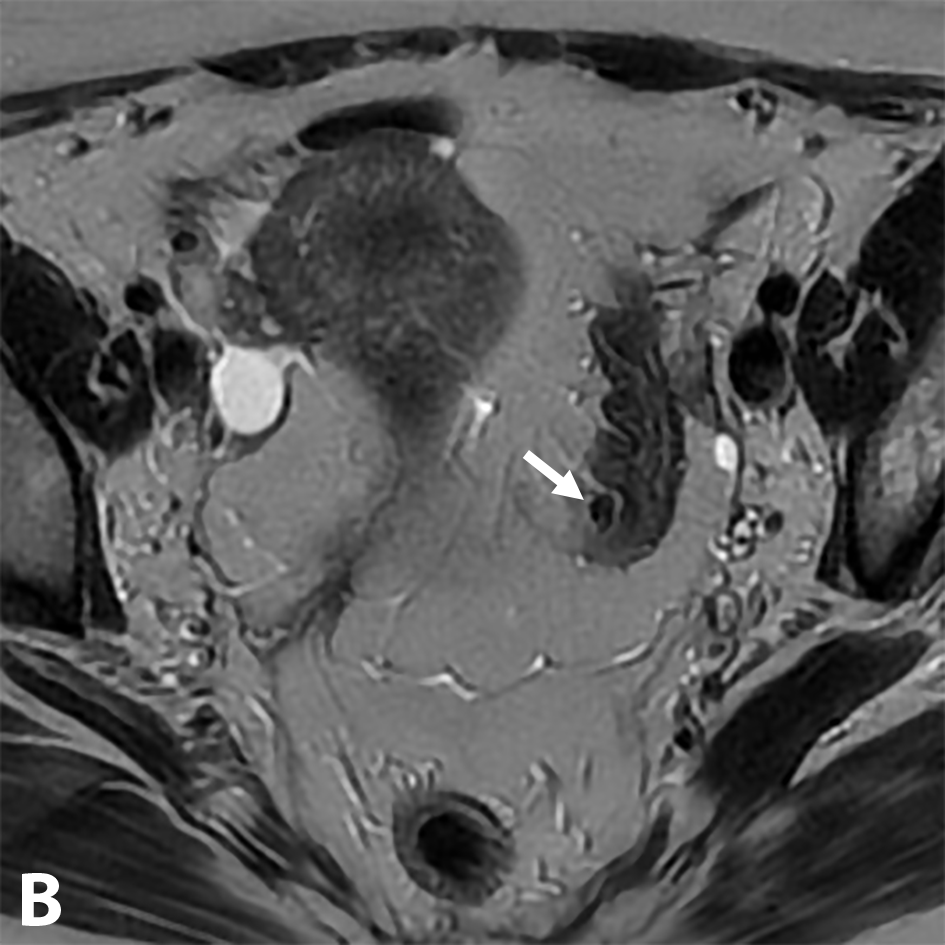


**Figure 11** - Sigmoid diverticula filled with feces in a 34-year-old woman with chronic pelvic pain.

(a) Axial T1-W fat-suppressed and (b) axial T2-W MR images show an outpouching colonic diverticulum with a small herniation filled with T1-hyperintense and T2-hypointense content in the lumen of the diverticula (arrows) referring to a stercoral concretion.


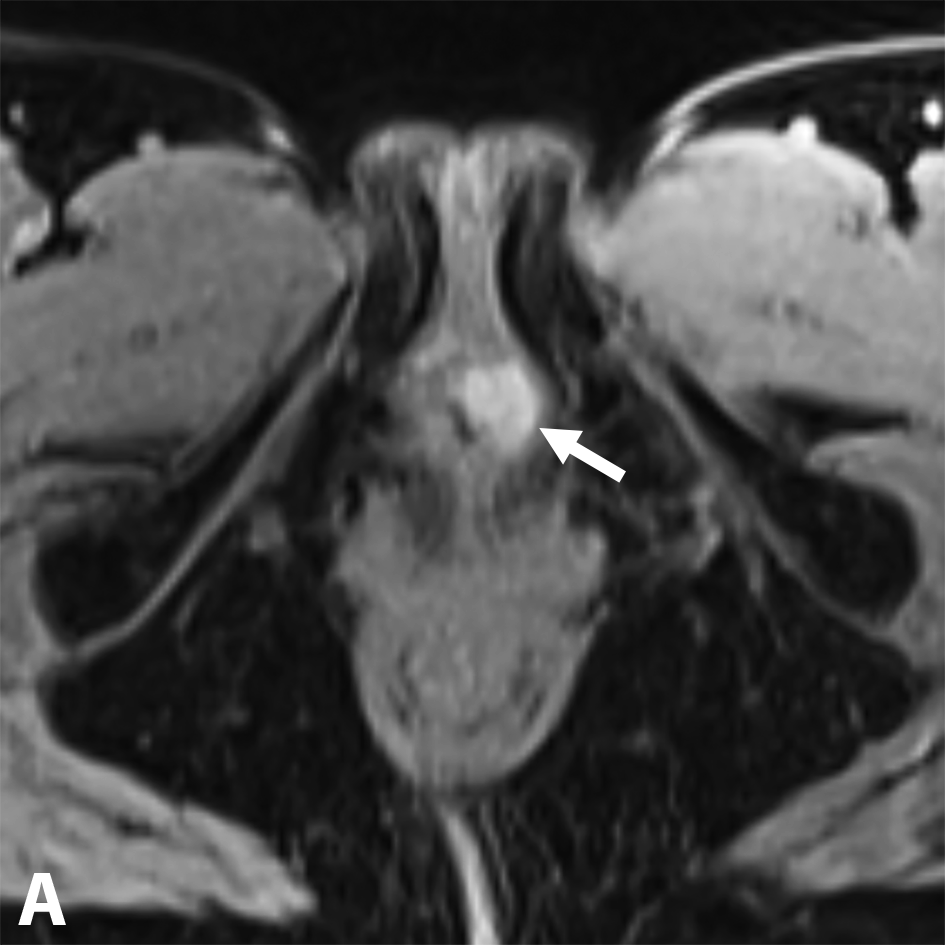

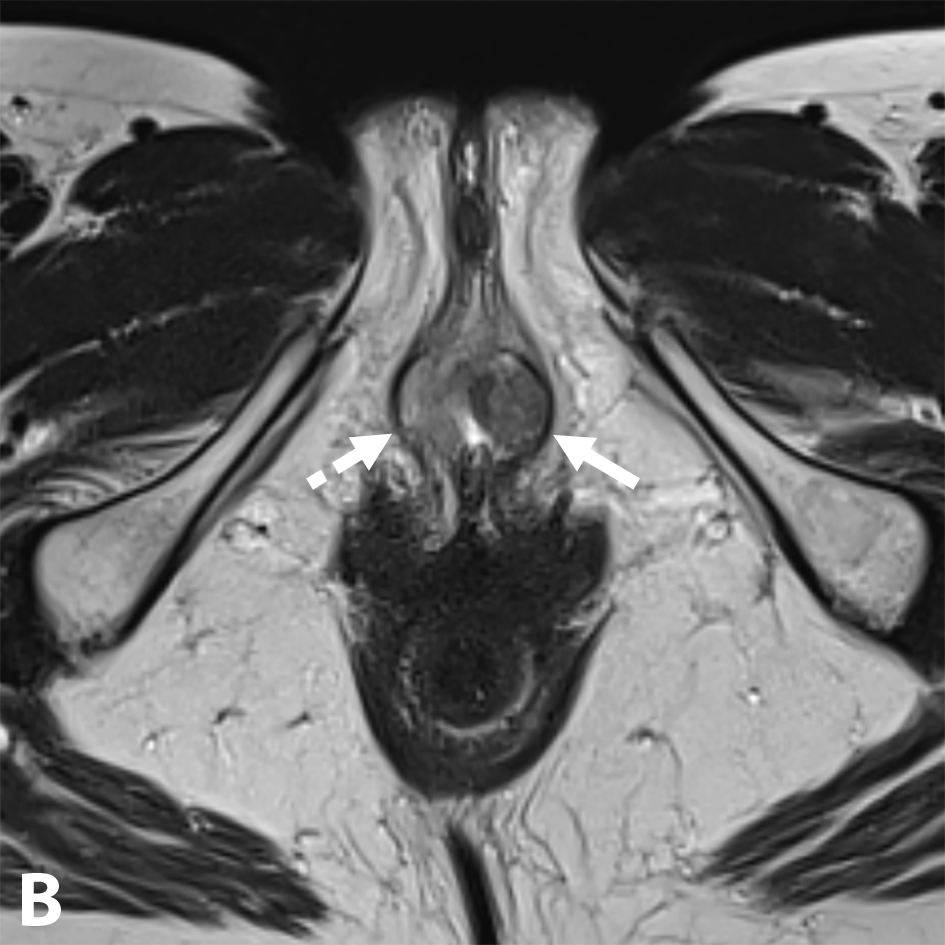

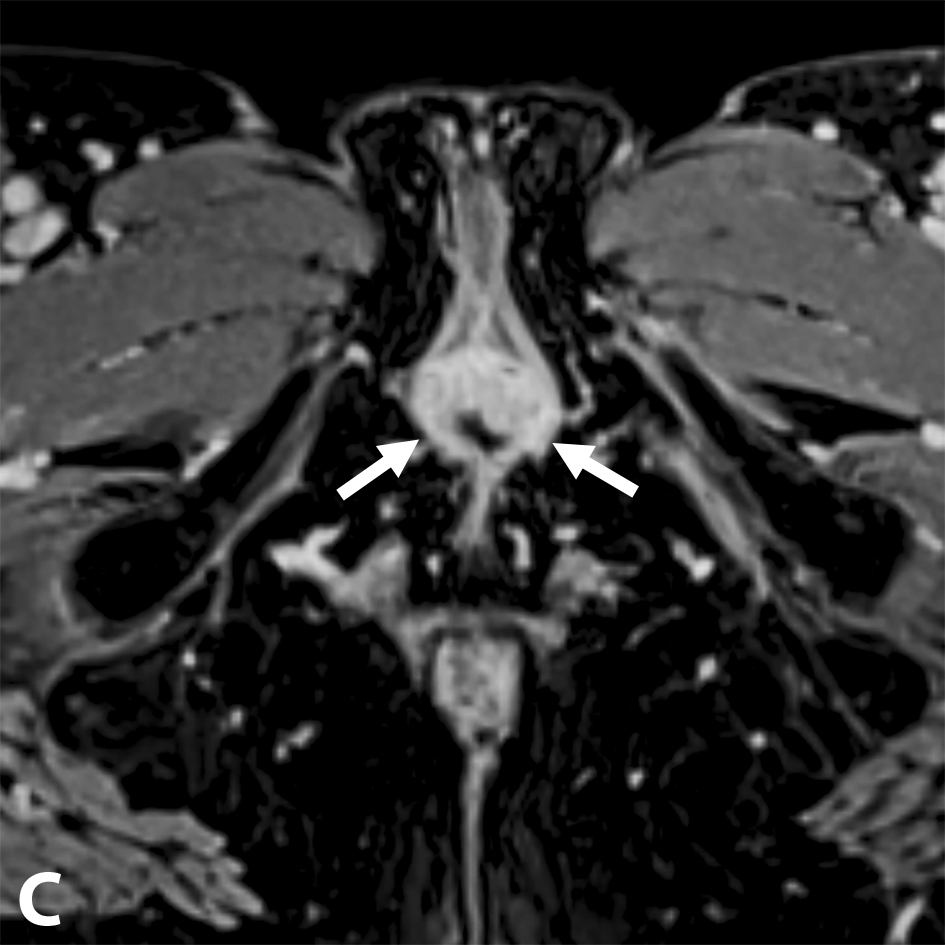


**Figure 12** - Vulvar melanoma in a 46-year-old patient.

(a) Axial fat-suppressed T1-W image shows a hyperintense oval mass (arrow) in the left inner lip (labia minora) of the vulva.

(b) Axial T2-W image shows intermediate signal intensity of the left part of the mass (thin arrow) and a slightly more irregular intermediate signal intensity of the right inner lip infiltration (dashed arrow).

(c) Axial fat-suppressed contrast enhanced subtracted T1-W image shows extensive enhancement of the solid tumor in both labia minora (arrows).
